# SUMO promotes DNA repair protein collaboration to support alternative telomere lengthening in the absence of PML

**DOI:** 10.1101/gad.351667.124

**Published:** 2024-07-01

**Authors:** Rongwei Zhao, Meng Xu, Xiaoyang Yu, Anne R. Wondisford, Rachel M. Lackner, Jayme Salsman, Graham Dellaire, David M. Chenoweth, Roderick J. O'Sullivan, Xiaolan Zhao, Huaiying Zhang

**Affiliations:** 1 Department of Biology, Carnegie Mellon University, Pittsburgh, Pennsylvania 15213, USA;; 2 Department of Pharmacology and Chemical Biology, University of Pittsburgh Medical Center Hillman Cancer Center, University of Pittsburgh, Pittsburgh, Pennsylvania 15213, USA;; 3 Department of Chemistry, University of Pennsylvania, Philadelphia, Pennsylvania 19014, USA;; 4 Department of Pathology, Dalhousie University, Halifax, Nova Scotia B3H 4R2, Canada;; 5 Molecular Biology Program, Memorial Sloan Kettering Cancer Center, New York, New York 10065, USA

**Keywords:** ALT cancer, condensates, DNA repair, PML body, phase separation, Rad52, SUMO

## Abstract

The alternative lengthening of telomeres (ALT) pathway maintains telomere length in a significant fraction of cancers that are associated with poor clinical outcomes. A better understanding of ALT mechanisms is therefore necessary for developing new treatment strategies for ALT cancers. SUMO modification of telomere proteins contributes to the formation of ALT telomere-associated PML bodies (APBs), in which telomeres are clustered and DNA repair proteins are enriched to promote homology-directed telomere DNA synthesis in ALT. However, it is still unknown whether—and if so, how—SUMO supports ALT beyond APB formation. Here, we show that SUMO condensates that contain DNA repair proteins enable telomere maintenance in the absence of APBs. In PML knockout ALT cell lines that lack APBs, we found that SUMOylation is required for manifesting ALT features independent of PML and APBs. Chemically induced telomere targeting of SUMO produces condensate formation and ALT features in PML-null cells. This effect requires both SUMOylation and interactions between SUMO and SUMO interaction motifs (SIMs). Mechanistically, SUMO-induced effects are associated with the accumulation of DNA repair proteins, including Rad52, Rad51AP1, RPA, and BLM, at telomeres. Furthermore, Rad52 can undergo phase separation, enrich SUMO at telomeres, and promote telomere DNA synthesis in collaboration with the BLM helicase in a SUMO-dependent manner. Collectively, our findings suggest that SUMO condensate formation promotes collaboration among DNA repair factors to support ALT telomere maintenance without PML. Given the promising effects of SUMOylation inhibitors in cancer treatment, our findings suggest their potential use in perturbing telomere maintenance in ALT cancer cells.

## Abstract (precis)

In this study, Zhao et al. report that SUMOylation induces phenotypes associated with the alternative lengthening of telomeres (ALT), independent of PML and PML bodies (APBs). SUMO forms condensates with DNA repair factors, is recruited to telomeres, and, with BLM helicase, promotes telomeric DNA synthesis, highlighting the potential of SUMOylation inhibitors in controlling ALT in cancers.

To sustain continuous proliferation, cancer cells must maintain their telomeres by telomerase reactivation or by alternative lengthening of telomeres (ALT) (Henson et al. 2002; Varley et al. 2002; Dilley and Greenberg 2015). An estimated 10%–15% of cancer types use ALT, and these are often associated with poor survival outcomes (Yeager et al. 1999; Dilley and Greenberg 2015; Zhang and Zou 2020). Past studies have established that telomere synthesis in ALT is achieved by break-induced replication (BIR), a specialized homology-directed repair (HDR) mechanism (Cho et al. 2014; Dilley et al. 2016; Roumelioti et al. 2016; Verma et al. 2019). ALT has been shown to use two BIR pathways mediated by either the recombination protein Rad52 (Min et al. 2017; Zhang et al. 2019) or the repair protein Rad51AP1 (Barroso-González et al. 2019; Verma et al. 2019; Zhang et al. 2019; Yadav et al. 2022). In addition, both of the pathways are enabled by the BLM helicase, which can process BIR intermediates (Zhang et al. 2019, 2021).

SUMOylation, the process by which the small ubiquitin modifier (SUMO) is conjugated to target proteins, has been shown on several occasions to have a major role in the faithful and efficient execution of ALT HDR (Potts and Yu 2007; Osterwald et al. 2015; Min et al. 2019; Loe et al. 2020). SUMOylation of telomere proteins such as TRF1 and TRF2 can promote the formation of ALT-specific promyelocytic leukemia (PML) bodies called APBs (ALT telomere-associated PML bodies) (Potts and Yu 2007; Brouwer et al. 2009; Chung et al. 2011). As the name suggests, APB formation also depends on the PML protein, which both is SUMOylated and contains SUMO interaction motifs (SIMs) (Kamitani et al. 1998; Shen et al. 2006; Sahin Umut et al. 2014). SIMs bind noncovalently to SUMOs attached to the PML proteins, providing multivalent interactions between PML proteins to drive phase separation and promote the formation of PML bodies (Banani et al. 2016). At ALT telomeres, the multivalent SUMO–SIM interactions between PML and telomere proteins drive APB formation via phase separation (Zhang et al. 2020). It is thought that APBs facilitate ALT by enriching the telomere clusters and DNA repair proteins within the same space (Draskovic et al. 2009; Zhang et al. 2021). Despite the critical roles of APBs in ALT, a recent study examining cancer cell lines that rely on ALT for telomere maintenance reported that while PML loss eliminated APBs and reduced ALT, cells remained viable for months (Loe et al. 2020). This finding suggested the possibility of APB-independent telomere maintenance in PML knockout (KO) cells. However, the functional importance and mechanistic contribution of SUMO to telomeres in PML KO cells are unknown.

Here, by examining PML KO ALT-positive U2OS cells, which lack APBs, we observed that SUMO remains accumulated at telomeres in the absence of PML and APBs. Importantly, we found that telomere breaks induced by the FokI nuclease fused to the telomere protein TRF1 can induce two key ALT features in PML KO cells; namely, telomere clustering and telomeric DNA synthesis. This result provides evidence that ALT activities can occur upon inducing telomere damage, even in the absence of PML and APBs. We further applied two experimental strategies not previously used in ALT studies. These include the application of SUMOylation inhibitors and chemically induced protein targeting, both of which allow live-cell imaging and prevent toxicity caused by constitutive telomere targeting. SUMO inhibitor studies revealed that SUMOylation is required for ALT features induced by telomere damage with TRF1-FokI in PML KO cells. This result argues for a role of SUMOylation in ALT that is distinct from its function in APB formation. By targeting SUMO to telomeres with chemically induced protein dimerization, we observed the induction of ALT features in the absence of PML, which requires both SUMOylation and SUMO–SIM interactions. Finally, we show that targeting SUMO and Rad52 to telomeres in PML KO cells induces the signature of phase separation and leads to the enrichment of both proteins at telomeres. Targeting both SUMO and Rad52 promotes telomere clustering and telomere synthesis in PML KO cells by enhancing the accumulation of the BLM helicase at telomeres. Collectively, our data provide several lines of evidence to support the conclusion that SUMO, in collaboration with Rad52, can promote ALT telomere maintenance by facilitating BLM function in the absence of PML and APBs.

## Results

### ALT phenotypes can be induced with telomere breaks in PML KO cells

In pursuing a mechanistic understanding of how SUMOylation contributes to ALT beyond APB formation, we performed comparative studies in ALT-positive U2OS cells and PML knockout (KO) U2OS cells that are devoid of APBs (Loe et al. 2020). The absence of PML nuclear bodies in PML KO cells was confirmed by immunofluorescence staining (Supplemental Fig. S1A). RT-PCR assessment of telomerase components suggested that both the PML KO and the WT U2OS cells did not reactivate telomerase (Supplemental Fig. S1B). As seen previously (Loe et al. 2020), the growth of U2OS cells was largely unaffected by the loss of PML. We conducted several assays to evaluate the role of SUMOylation in ALT in the absence of APBs and PML.

**Figure 1.**
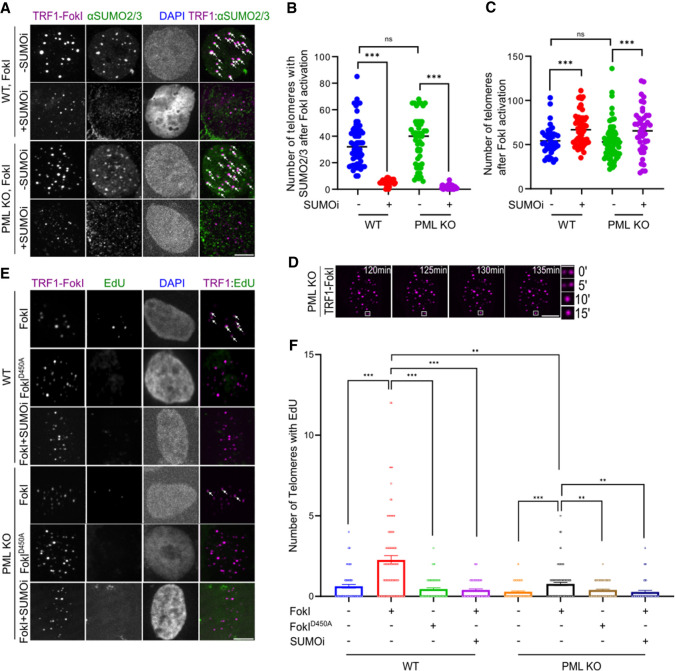
SUMO inhibition diminishes FokI-induced ALT features in PML KO cells. (*A–C*) Representative images (*A*), quantification of SUMO2/3 localization at telomeres (*B*), and telomere numbers (*C*) in WT U2OS and PML knockout (KO) U2OS cells expressing mCh-TRF1-FokI. Cells were treated with 4-hydroxyestradiol (4-OHT) for 6 h to allow FokI to translocate into the nucleus. Cells were further treated with 1 μM SUMO inhibitor (SUMOi) for 2 days or were untreated. Each dot represents one cell, three independent experiments, >60 cells per group. White arrows indicate SUMO2/3 colocalizations at telomeres. (*D*) Live-cell imaging of PML KO cells expressing mCh-TRF1-FokI, which induces telomere-specific DNA damage. Zoomed-in images at the *right* show a telomere clustering event. (*E*,*F*) Representative images (*E*) and quantification (*F*) of EdU staining at telomeres after 6 h of FokI induction with or without SUMOi treatment. White arrows indicate EdU signals at telomeres. Each dot represents one cell, three independent experiments, >75 cells per group. Scale bars: zoomed-in images in *D*, 1 μm; other images, 5 μm.

We first examined ALT features induced by telomere-specific DNA breaks, which can be generated by fusing the FokI nuclease to the telomeric protein TRF1, thereby targeting it to telomeres (Cho et al. 2014; Dilley et al. 2016). While previous studies have used this system in PML-proficient cells, we applied it to PML KO U2OS cells to query whether telomere breaks can induce ALT features without PML and APBs and whether these features require SUMO. First, we assessed whether SUMO was localized to telomeres after FokI induction. Immunofluorescence imaging revealed that the TRF1-FokI fusion increased the accumulation of both SUMO1 and SUMO2/3 (SUMO2 and SUMO3 were detected by the same antibody) at telomeres in PML KO cells, whereas the nuclease-dead mutant fusion TRF1-FokI-D450A did not (Fig. 1A,B; Supplemental Fig. S1C,D,H,I). The level of telomeric SUMO in PML KO cells was similar to that seen in the control PML-proficient cells (Fig. 1B; Supplemental Fig. S1I). In addition, MMS21 and PIAS4, two SUMO ligases important for ALT (Potts and Yu 2005, 2007; Brouwer et al. 2009; Chung et al. 2011; Zhang et al. 2021), were found at telomeres in both PML KO and PML-containing cells (Supplemental Fig. S1F,G). These data show that SUMO and SUMO ligase enrichment at ALT telomeres occurs robustly in response to telomere breaks without PML and APBs.

Significantly, TRF1-FokI induced telomere clustering in PML KO cells, whereas TRF1-FokI-D450A did not, as evidenced by a reduction in telomere numbers detected by telomere DNA fluorescence in situ hybridization (FISH) (Supplemental Fig. S1E). The degree of telomere clustering in the PML KO cells was similar to that observed in the PML-proficient control cells (Fig. 1A,C). Live-cell imaging further captured the process of telomere clustering after FokI induction in PML KO cells (Fig. 1D; Supplemental Movie S1), as reported in PML-proficient cells (Cho et al. 2014). These data show that telomere breaks can induce robust telomere clustering, bypassing the requirement for PML and APBs.

Next, we measured nascent telomeric DNA synthesis by assessing EdU incorporation at telomeres in non-S-phase cells (Cho et al. 2014). Telomeric DNA synthesis was increased in PML KO cells after TRF1-FokI induction (Fig. 1E,F). Unlike SUMO accumulation (Fig. 1B) and telomere clustering (Fig. 1C), telomeric DNA synthesis was less pronounced in PML KO cells than in PML-proficient control cells, suggesting that PML and APBs are more important for telomeric DNA synthesis than for SUMO enrichment and telomere clustering. In conclusion, our data are consistent with an established role of PML and APBs in ALT cells (O'Sullivan et al. 2014) and further reveal that telomere breaks can induce three key ALT features in the absence of PML and APBs: localization of SUMO to telomeres, telomere clustering, and telomeric DNA synthesis.

### SUMOylation is required for damage-induced ALT phenotypes in PML KO cells

We asked whether SUMOylation is required for telomere clustering and telomere DNA synthesis independent of PML and APBs. To this end, we used a recently developed small molecule inhibitor (ML-792; SUMOi) that inactivates the SUMO E1 enzyme to downregulate SUMOylation (He et al. 2017). Treatment with ML-792 using a previously established scheme to reduce 90% of cellular SUMOylation (He et al. 2017) efficiently decreased SUMO1 and SUMO2/3 levels at telomeres in both PML-null and PML-containing cells (Fig. 1A,B; Supplemental Fig. S1H,I), confirming the effectiveness of the inhibitor. Significantly, SUMOi treatment decreased telomere clustering upon TRF1-FokI induction in both types of cells (Fig. 1C). In addition, upon SUMOi treatment, both types of cells showed decreased telomeric DNA synthesis upon TRF1-FokI induction, reaching levels similar to those of cells without TRF1-FokI or with TRF1-FokI-D450A (Fig. 1E,F). These results suggest that upon induction of telomere breaks, SUMOylation aids ALT telomere clustering and telomere DNA synthesis in the absence of PML and APBs.

### SUMOylation is required for endogenous ALT features in PML KO cells

**Figure 2.**
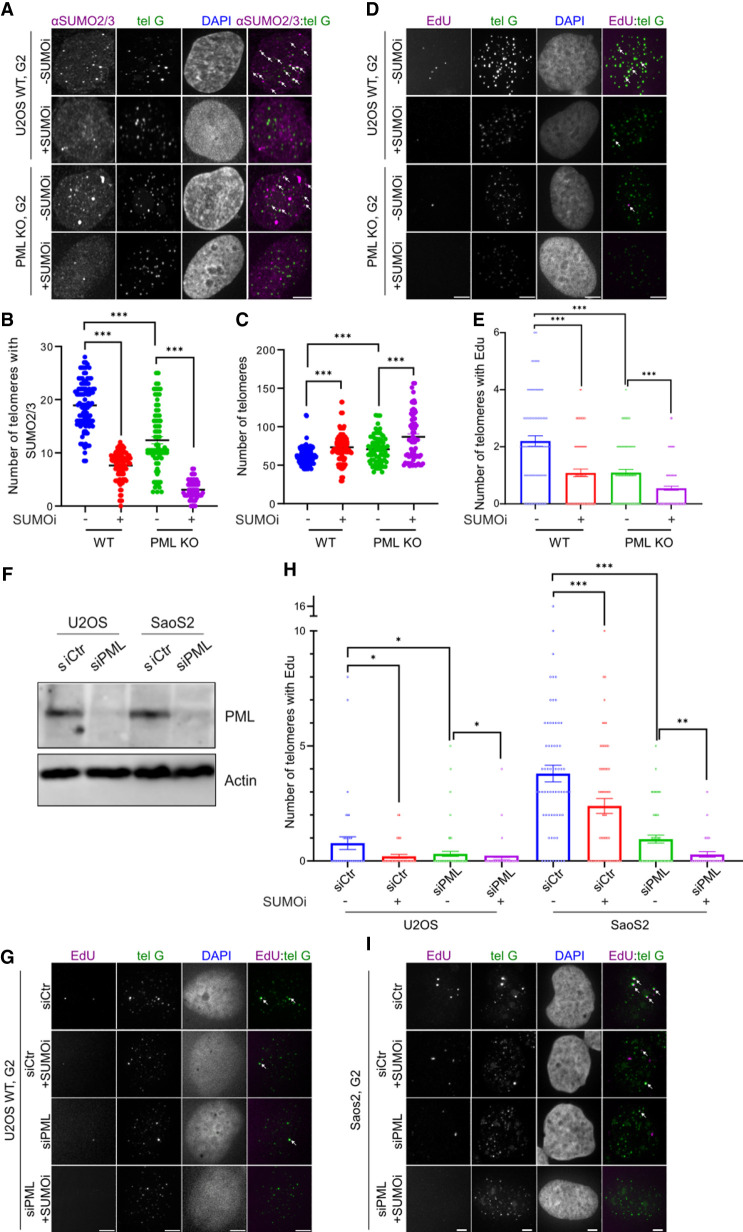
SUMO inhibition reduces endogenous ALT features in PML-null cells. (*A–C*) Representative images (*A*), quantification of SUMO2/3 localization at telomeres (*B*), and quantification of telomere numbers (*C*) in G2-arrested WT and PML KO U2OS cells with or without SUMOi treatment. Each dot represents one cell, three independent experiments, >45 cells per group. (*D*,*E*) Representative images (*D*) and quantification (*E*) of telomeres with EdU staining in G2-arrested WT and PML KO cells with or without SUMOi treatment. White arrows indicate EdU signals at telomeres. Each dot represents one cell, three independent experiments, >80 cells per group. (*F*) Western blot of PML after transfecting control siRNA and siPML in U2OS and Saos2 cells. (*G*–*I*) Representative images (*G*,*I*) and quantification (*H*) of EdU foci at telomeres in U2OS and Saos2 cells after transfecting control siRNA or siPML with or without SUMOi. White arrows indicate EdU signals at telomeres. Each dot represents one cell, three independent experiments, >37 cells per group. Scale bars, 5 μm.

Prompted by these observations, we also examined how endogenous ALT is affected by SUMOylation in the presence and absence of APBs and PML. To this end, we arrested cells in the G2 phase when ALT is most active (Zhang et al. 2019). First, immunofluorescence imaging detected accumulation of SUMO1 and SUMO2/3 at telomeres in PML KO cells but at a level significantly lower than in PML-containing control cells (Fig. 2A,B; Supplemental Fig. S2A,B,E,F). As expected, treating both types of cells with SUMOi significantly reduced telomeric SUMO1/2/3 signals (Fig. 2A,B; Supplemental Fig. S2A,B,E,F). The effectiveness of SUMOi treatment was also evident in the reduced numbers of APBs in PML-containing cells (Supplemental Figs. S1J,K, S2C,D), an effect that was anticipated based on the established role of SUMO in the formation of PML bodies and APBs (Zhong et al. 2000; Potts and Yu 2007; Chung et al. 2011; Hirano and Udagawa 2022).

Second, we assessed telomere clustering and found that PML KO reduced this feature as expected (Fig. 2C). Importantly, SUMOi treatment led to reduced telomere clustering in both PML KO and control WT cells (Fig. 2C). This result suggests that SUMOylation inhibition can reduce endogenous telomere clustering and that this effect can be seen in the absence of PML.

Third, we quantified nascent telomeric DNA synthesis based on EdU staining at telomeres. EdU foci at telomeres (colocalized with telomeres) were still present in PML KO cells but at a reduced level compared with the control PML-containing cells (Fig. 2D,E). Importantly, SUMOi treatment reduced the numbers of EdU foci at telomeres in both types of cells (Fig. 2D,E). These data agree with the established role of PML in ALT and further reveal that SUMO can contribute to endogenous ALT in the absence of PML.

These observations were confirmed by experiments with siRNA knockdown of PML in U2OS cells and another ALT-positive cell line, Saos2. PML knockdown efficiency was confirmed using Western blot in both cell types (Fig. 2F). Compared with control cells, knockdown of PML in both cell types reduced ALT features in the G2 phase, as described above for PML KO U2OS cells. These effects include reductions in (1) telomeric localization of all three SUMO isoforms, (2) telomere clustering, and (3) telomeric DNA synthesis (Fig. 2G–I; Supplemental Fig. S3A–K). Also, as seen in PML KO cells, SUMOi treatment further diminished ALT features in both U2OS and Saos2 cells treated with siPML (Fig. 2G–I; Supplemental Fig. S3A–K). Endogenous ALT features under these conditions showed a dependence on SUMO similar to those derived from FokI-induced telomere breaks, suggesting that SUMOylation can contribute to ALT features in the absence of PML and APBs.

### Targeting SUMO to telomeres induces signatures of phase separation and ALT phenotypes in PML KO cells

**Figure 3.**
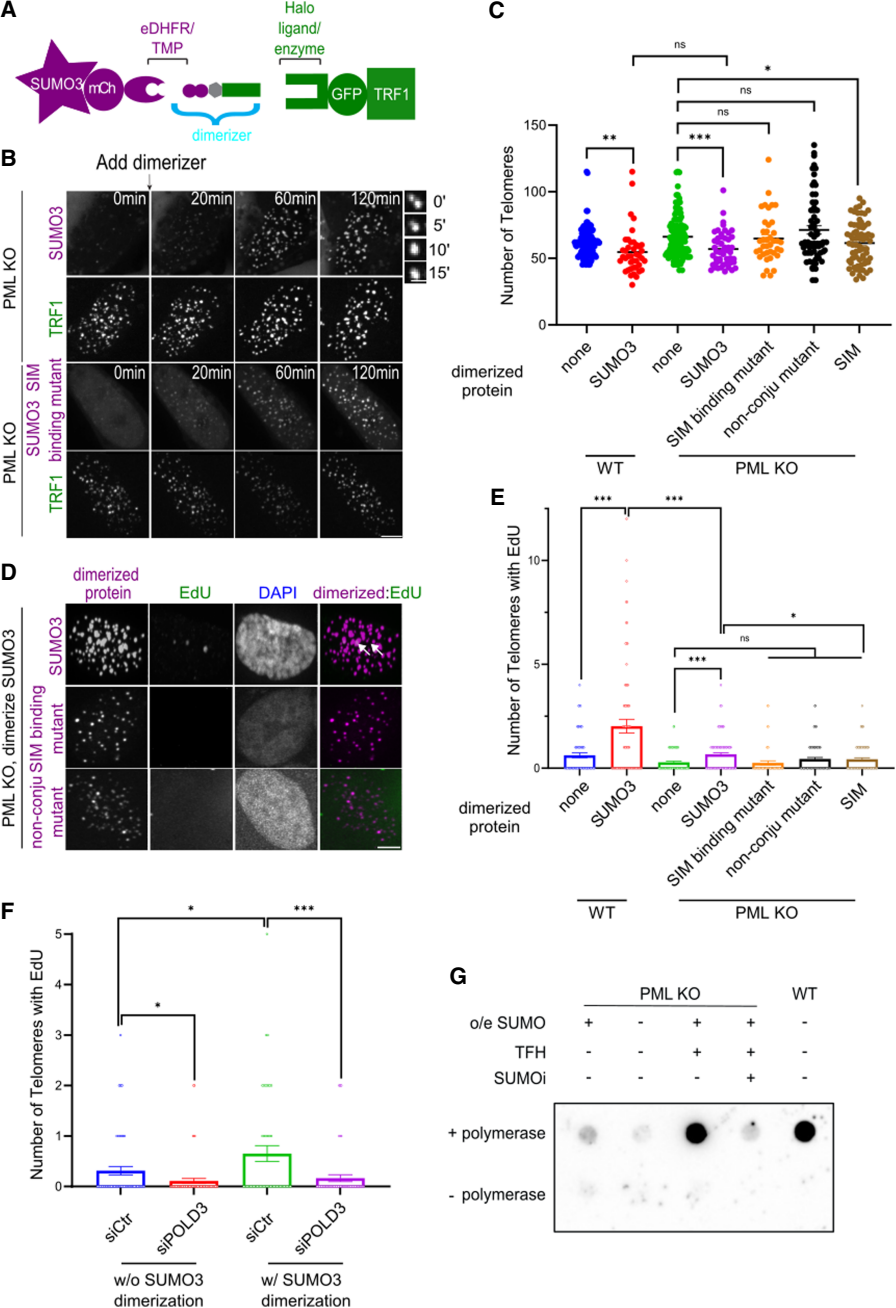
SUMO enrichment induces signatures of phase separation and ALT phenotypes in PML KO cells. (*A*) Dimerization schematic showing that SUMO3 is fused to mCherry and eDHFR, and that TRF1 is fused to GFP and the 3xHalo enzyme. The dimerizer TMP (trimethoprim)–fluorobenzamide–Halo ligand (TFH) can interact with eDHFR and the Halo enzyme to dimerize SUMO3 to TRF1. (*B*) Representative images of PML KO U2OS cells after dimerizing mCh-eDHFR-SUMO3 or SUMO3 SIM binding mutants to 3xHalo-GFP-TRF1 after the first time point. Zoomed-in images at the *right* show the fusion of TRF1 foci after dimerizing SUMO3. (*C*) Quantification of telomere number after dimerizing SUMO3, SUMO3 SIM-interacting mutants, SUMO3 nonconjugable mutants, and SIM to telomeres for 6 h. Each dot represents one cell, three independent experiments, >41 cells per group. (*D*,*E*) Representative images (*D*) and quantification (*E*) of EdU staining for newly synthesized telomere DNA with or without dimerizing SUMO3, SUMO3 mutants, and SIM to telomeres in PML KO cells. Telomeres were visualized by FISH (images not shown). White arrows indicate EdU foci at telomeres. Each dot represents one cell, three independent experiments, >54 cells per group. (*F*) Quantification of EdU staining for newly synthesized telomere DNA after transfecting control siRNA or siPOLD3 for 2 days, with or without dimerizing SUMO3, to telomeres in PML KO cells. Each dot represents one cell, three independent experiments, >50 cells per group. (*G*) C-circle dot blot for U2OS and PML KO cells after transfecting SUMO3 with or without adding dimerizer for 2 days. Scale bars: zoomed images in *B*, 1 μm; other images, 5 μm.

We have previously shown that SUMO-mediated phase separation induces telomere clustering in PML-proficient U2OS cells (Zhang et al. 2020). We asked whether enriching SUMO at telomeres in PML KO cells could have the same effects. To this end, we used an inducible protein dimerization system that can transiently and effectively recruit proteins of interest to specific genomic loci (Zhao et al. 2021; Lackner et al. 2022). In our setup, each SUMO isoform (SUMO1, SUMO2, and SUMO3) was fused to mCherry and the eDHFR protein, while the telomere protein TRF1 was fused to GFP and the 3xHalo enzyme (Fig. 3A). The addition of the chemical dimerizer TMP (trimethoprim)–fluorobenzamide–Halo ligand (TFH), which binds to both eDHFR and the Halo enzyme, can induce interaction of the two fusion proteins. This technique can minimize toxicity associated with constitutive SUMO enrichment at telomeres and is compatible with live-cell imaging to examine ALT features in real time.

We first confirmed the successful recruitment of each SUMO isoform to TRF1-GFP-marked telomeres upon the addition of the dimerizer TFH in the PML KO cells (Fig. 3B). Both SUMO-mCherry and TRF1-GFP formed bright and round foci and showed high degrees of colocalization (Fig. 3B; Supplemental Fig. S4A; Supplemental Movies S2–S4). These foci showed fusion behaviors characteristic of phase-separated condensates (Fig. 3B; Supplemental Fig. S4A; Supplemental Movies S2–S4). Moreover, telomere clustering was enhanced after dimerizer addition in PML KO cells, as determined by the reduction of telomere numbers in treated versus nontreated cells (Supplemental Fig. S4B). The increased telomere clustering was also confirmed with telomere DNA FISH (Fig. 3C; Supplemental Fig. S4C,D). Recruiting SUMO isoforms to telomeres also increased EdU staining at telomeres in non-S-phase cells (Fig. 3D,E; Supplemental Fig. S4E,F). In addition, the observed EdU signals require POLD3, an enzyme that catalyzes ALT telomere synthesis (Dilley et al. 2016), since the levels of EdU signal were reduced upon POLD3 knockdown (Fig. 3F; Supplemental Fig. S4H). Furthermore, telomere foci containing EdU were larger and brighter than those without, raising the possibility that telomeric DNA synthesis is favored at larger condensates (Supplemental Fig. S4G). Recruiting SUMO3 to telomeres in PML KO cells also led to increased C-circle levels, another ALT marker (Henson et al. 2009). Treatment with SUMOi eliminated this increase (Fig. 3G). These observed effects of dimerizer-induced SUMO recruitment to telomeres provide evidence for PML- and APB-independent formation of phase-separated condensates containing SUMO and several key ALT features, including telomere clustering, telomeric DNA synthesis, and C-circle formation.

For all examined effects described above, the three SUMO isoforms behaved similarly. Furthermore, the recruitment of one isoform enriched the others at telomeres. As SUMO inhibition reduced such mutual enrichment (Supplemental Fig. S5A,B), we conclude that isoform interdependency requires the conjugation of SUMOs to proteins. Focusing on SUMO3, we found that its recruitment to telomeres resulted in a similar level of telomere clustering in both PML-containing and PML KO cells (Fig. 3C) but more telomere DNA synthesis in PML-containing cells (Fig. 3E), suggesting that APBs are more relevant to ALT telomere synthesis than to telomere clustering. This notion was further supported by findings in HeLa cells, which are ALT-negative and lack APBs, as SUMO3 recruitment to telomeres in these cells failed to enable telomere DNA synthesis despite inducing low levels of APBs and telomere clustering (Supplemental Fig. S4I–M).

### SUMO–SIM interactions dictate the ALT features induced by SUMO targeting in PML KO cells

Previously, we found that recruiting SIM to telomeres in PML-containing cells induced APB formation via phase separation, and that this effect requires SUMO binding capacity (Zhang et al. 2020). We thus asked whether SUMO–SIM interactions could contribute to ALT in the absence of PML. To this end, we examined the consequences of targeting SIM to telomeres in PML KO cells using the chemical dimerizer system described above. Three effects were observed upon SIM recruitment to telomeres. First, SUMO isoforms were enriched at telomeres (Supplemental Fig. S5C–E). Second, TRF1-containing round droplets were found to fuse among themselves over time, suggesting phase separation behavior (Supplemental Fig. S5G,H). Third, telomere clustering was induced (Supplemental Fig. S5F,H), though to a lesser degree than when SUMO was targeted to telomeres (Fig. 3C). Interestingly, SIM targeting to telomeres did not induce telomere DNA synthesis (Fig. 3E), which was similar to what we reported in cells containing PML (Zhang et al. 2020) but different from what we observed when targeting SUMO to telomeres in PML KO cells (Fig. 3D,E). We suspect that this difference could be caused by variations in SUMO–SIM stoichiometries, as this feature affects the condensate composition (Banani et al. 2016; Ditlev et al. 2018). Regardless, the above findings support the notion that targeting SIM to telomeres can induce a subset of ALT features.

We then sought to investigate whether SUMO–SIM interactions are important for ALT features by using the dimerization system to target SUMO and SIM mutants incapable of SUMO–SIM binding to telomeres. First, we found that a SUMO3 variant mutated at its SIM binding site (FKIK mutated to FAAA) (Banani et al. 2016) did not induce signatures of phase separation (Fig. 3B; Supplemental Fig. S4B; Supplemental Movie S5) and reduced both telomere clustering (Fig. 3C) and telomere DNA synthesis (Fig. 3D,E). Second, recruiting a SIM mutant that cannot interact with SUMO (VIDL mutated to VADA) failed to generate TRF1-containing droplets or promote telomere clustering (Supplemental Fig. S5G,H). Third, recruiting a SUMO3 mutant that could not be conjugated to substrates (a di-Gly motif mutant) resulted in less telomere clustering (Fig. 3C) and less telomeric DNA synthesis (Fig. 3D,E). This is accompanied by a lack of enrichment of SUMO1/2 at telomeres (Supplemental Fig. S5A,B). These results are consistent with each other and suggest that protein SUMOylation and SUMO–SIM interaction contribute to SUMO-induced ALT features in PML KO cells.

### SUMO–SIM interactions sequester HR factor to telomeres in the absence of PML

SUMOylation enables the assembly of DNA repair protein networks in response to DNA damage via SUMO–SIM interactions (Psakhye and Jentsch 2012; Hu and Parvin 2014; Dhingra and Zhao 2019; Claessens et al. 2023). Thus, we hypothesized that DNA repair factors contributing to ALT and containing SUMOylation sites and/or SIMs could mediate SUMO–SIM-dependent effects on ALT. After surveying the literature, we tested three proteins containing at least one predicted and/or confirmed SUMOylation and SIM site. These include the BLM helicase (Ouyang et al. 2009; Min et al. 2019), Rad51AP1 (Barroso-González et al. 2019), and Rad52 (Sacher et al. 2006; Torres-Rosell et al. 2007; Silva et al. 2016; Verma et al. 2019). Whereas Rad52 and Rad51AP1 have been implicated in distinct ALT pathways (Kaminski et al. 2022; Yadav et al. 2022), BLM exhibits multifunctional activities in ALT DNA damage response initiation, DNA synthesis, HR intermediate dissolution, and APB formation (O'Sullivan et al. 2014; Min et al. 2019; Panier et al. 2019; Zhang et al. 2019; Loe et al. 2020; Jiang et al. 2024; Lee et al. 2024). Thus, the behavior of these proteins can help us understand how SUMO–SIM interactions affect both ALT pathways.

**Figure 4.**
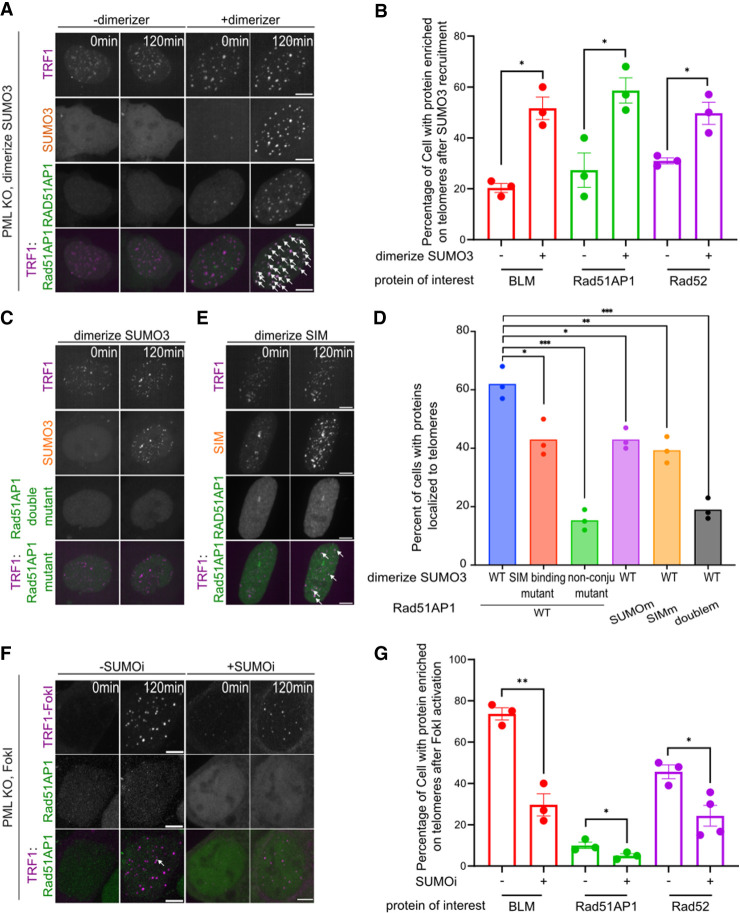
SUMO promotes DNA repair factor enrichment at telomeres independent of APBs. (*A*) Representative images of PML KO cells expressing mCherry-eDHFR-SUMO3, miRFP670-3xHalo-TRF1, and GFP-Rad51AP1 with or without adding dimerizer to dimerize SUMO3 to TRF1 at the indicated time points. (*B*) Quantification of the percentage of cells that have the indicated proteins enriched at telomeres after dimerizing SUMO3 to PML KO telomeres for 2 h. Each dot represents one experiment, three independent experiments, >35 cells in each group. (*C*) Representative images of PML KO cells expressing mCherry-eDHFR-SUMO3, miRFP670-3xHalo-TRF1, and GFP-Rad51AP1 SUMO/SIM double mutants after adding dimerizer at the indicated time point. (*D*) Quantification of cells that have the indicated proteins enriched to PML KO telomeres after 2 h of adding dimerizers. Each dot represents one experiment, three independent experiments, >28 cells per group. (*E*) Representative images of PML KO cells expressing mCherry-eDHFR-SIM, miRFP670-3xHalo-TRF1, and GFP-Rad51AP1 after adding dimerizer at the indicated time point. (*F*) Representative images of PML KO cells expressing mCh-TRF1-FokI and GFP-Rad51AP1 with treatment with 4-hydroxyestradiol (4-OHT) to induce DNA damage at the indicated time point, treated with or without 1 μM SUMOi for 2 days. White arrows indicate Rad51AP1 colocalizations with telomeres. (*G*) Quantification of PML KO cells that have the indicated proteins enriched at telomeres after inducing FokI-TRF1 and with or without 1 μM SUMOi treatment for 2 days. Each dot represents one experiment, three independent experiments, >30 cells per group. Scale bars, 5 μm.

We found that targeting SUMO3 to telomeres in PML KO cells induced enrichment of BLM, Rad51AP1, and Rad52 at telomeres (Fig. 4A,B; Supplemental Fig. S6A,B,I). Similar effects were seen when SIM was targeted to telomeres, though to a lesser degree (Fig. 4D,E; Supplemental Fig. S6D,G,H). The differences here are consistent with the relatively weaker effects of SIM on inducing ALT features described above (Fig. 3C,E). In addition, recruiting SIM enriched more Rad52 and Rad51AP1 than BLM (Supplemental Fig. S6D,G,H), likely due to the different affinity of these proteins to SIM. These observations suggest that the three key ALT players can be recruited to telomeres by increasing SUMO abundance in the absence of PML and APBs.

Notably, lower enrichment of BLM, Rad51AP1, and Rad52 at telomeres was observed when nonconjugatable SUMO1 or SUMO3 was used or when SUMOi was applied, indicating that SUMOylation is required for enrichment (Fig. 4D; Supplemental Fig. S6C–F). Reduced levels of the three repair proteins were found at telomeres when a SUMO3 mutant defective in SIM binding was used, indicating that this effect also requires the SUMO–SIM interaction (Fig. 4D; Supplemental Fig. S6C–H). The enrichment of Rad51AP1 was consistently diminished when its SUMOylation and SIM sites were mutated, either separately or together (Fig. 4C,D). These data support the conclusion that SUMOylation and SUMO–SIM interactions contribute to telomere enrichment of BLM, Rad51AP1, and Rad52 in the absence of PML and APBs.

Next, we used the TRF1-FokI-induced telomere break system described above in PML KO U2OS cells to assess the contribution of SUMOylation to telomeric localization of BLM, Rad51AP1, and Rad52. After induction with TRF1-FokI, localization of BLM, Rad52, and Rad51AP1 increased at telomeres (Supplemental Fig. S7C). This effect was not observed with the catalytically dead mutant of FokI, suggesting that DNA breaks promote the recruitment of these proteins to ALT telomeres without PML. Notably, their recruitment required SUMOylation, as recruitment levels were reduced upon SUMOi treatment (Fig. 4F,G; Supplemental Fig. S7A,B). This result is consistent with that obtained with SUMO targeting (Fig. 4D; Supplemental Fig. S6C–F).

We also assessed the ssDNA binding complex RPA and the recombinase Rad51, which have been implicated in ALT and contain SUMOylation sites and SIMs (Shima et al. 2013; Cho et al. 2014; Maréchal and Zou 2015; Cappadocia et al. 2021; Lezaja et al. 2021; Zhu et al. 2023). Telomeric enrichment was seen for RPA but not Rad51 after TRF1-FokI activation or SUMO3 targeting in PML KO cells, and SUMOi treatment eliminated the RPA enrichment (Supplemental Fig. S7D–H). This result is consistent with the notion that SUMO contributes to the telomeric localization of some HR factors, such as Rad51AP1, BLM, Rad52, and RPA, in the absence of PML and APBs.

### Rad52 recruitment induces SUMO enrichment and signatures of phase separation in PML KO cells

**Figure 5.**
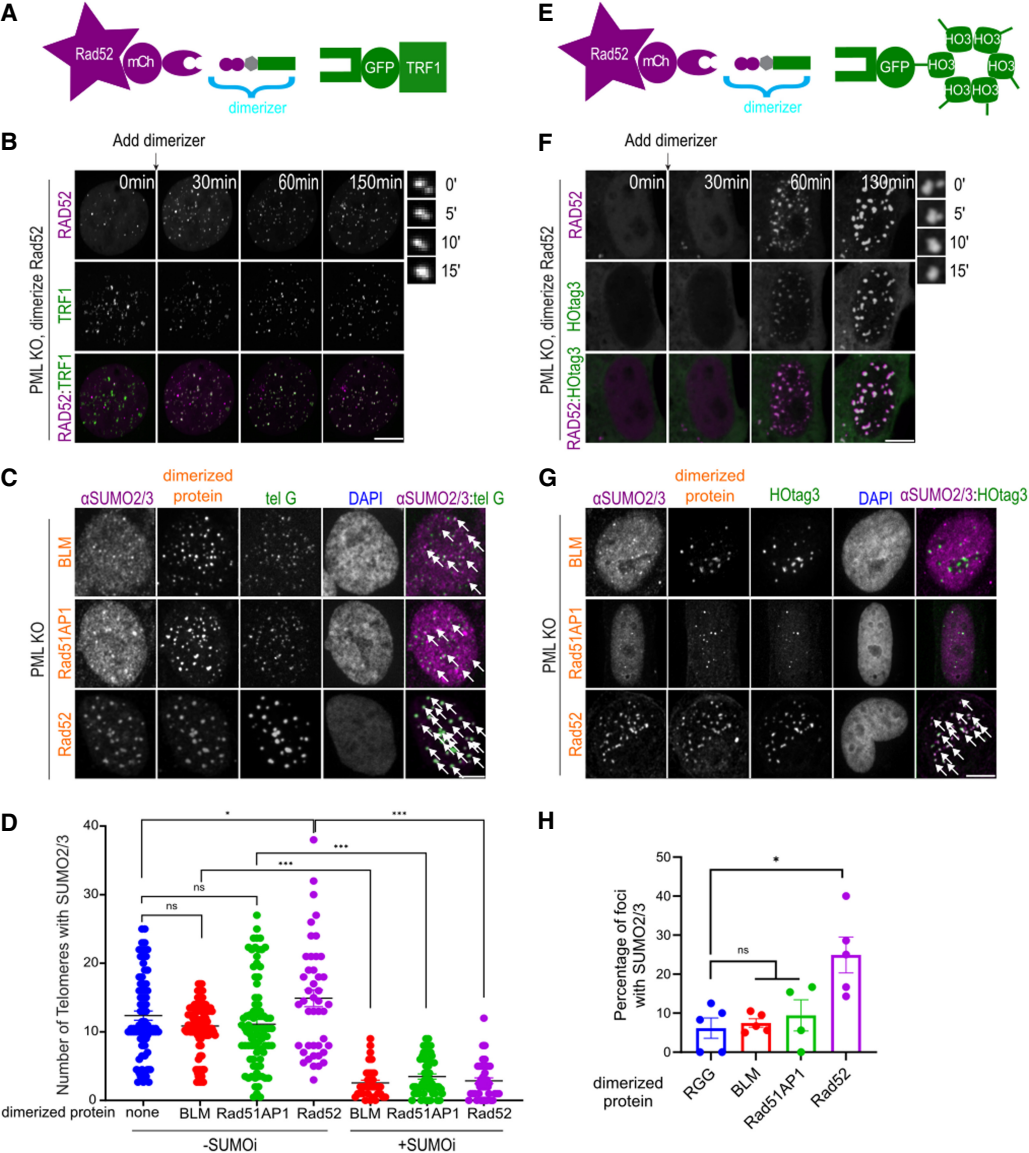
Rad52 recruitment induces phase separation and enriches SUMO. (*A*) Dimerization schematic showing that Rad52/BLM/Rad51AP1 is fused to mCherry and eDHFR, and that TRF1 is fused to GFP and the 3xHalo enzyme. (*B*) Representative images of PML KO cells after dimerizing mCh-eDHFR-Rad52 to 3xHalo-GFP-TRF1 at the indicated time points. Zoomed-in images at the *right* show a fusion event of TRF1 foci. (*C*,*D*) Representative images (*C*) and quantification (*D*) of SUMO2/3 localization at telomeres in PML KO cells expressing mCh-eDHFR-BLM/Rad51AP1/Rad52 and 3xHalo-TRF1 after adding the dimerizer for 6 h with or without 1 μM SUMOi for 2 days. White arrows indicate SUMO2/3 localization at telomeres. Each dot represents one cell, three independent experiments, >53 cells per group. (*E*) Dimerization schematic showing that Rad52/BLM/Rad51AP1 is fused to mCherry and eDHFR, and that HOtag3 is fused to GFP and the 3xHalo enzyme. (*F*) Representative images of PML KO cells expressing mCh-eDHFR-Rad52 and 3xHalo-GFP-HOtag3 after adding the dimerizer to induce dimerization at the indicated time points. Zoomed-in images at the *right* show a fusion event over time. (*G*,*H*) Representative images (*G*) and quantification (*H*) of SUMO2/3 localization in foci in PML KO cells after dimerizing mCh-eDHFR-BLM, Rad51AP1, Rad52, or RGG to 3xHalo-GFP-HOtag3 for 3 h. Each dot represents one experiment, three independent experiments, >24 cells per group. Scale bars: zoomed images in *B*,*F*, 1 μm; other images, 5 μm.

Next, we asked whether directly targeting BLM, Rad52, and Rad51AP1 to telomeres can promote ALT features. To this end, Rad52, Rad51AP1, and BLM were individually recruited to telomeres in PML-null U2OS cells using the dimerizer system described above (Fig. 5A). Live-cell imaging of the GFP-TRF1 foci, which represent telomeres, showed an increase in intensity and a decrease in numbers over time, suggesting the formation of telomere-containing phase-separated condensates (Fig. 5B; Supplemental Fig. S8A–E; Supplemental Movies S6–S8). Importantly, all three tested proteins formed bright and round foci that showed fusion behaviors characteristic of phase-separated condensates (Fig. 5B; Supplemental Fig. S8A–E; Supplemental Movies S6–S8). In all three cases, telomeres became clustered as a result of condensate fusion, as confirmed by telomeric DNA FISH (Supplemental Fig. S8J). These effects resembled those observed upon targeting SUMO or SIM to telomeres as described above (Fig. 3C). Thus, targeting BLM, Rad52, and Rad51AP1 to telomeres in PML KO cells can induce nuclear structures with features of phase-separated condensates that contain both telomeres and the DNA repair proteins in a way that is similar to APBs.

Among the three proteins examined, only telomere targeting of Rad52 led to increased levels of SUMO at telomeres (Fig. 5C,D; Supplemental Fig. S8F,I). Furthermore, SUMOylation is required for Rad52-induced SUMO enrichment, since this effect was eliminated upon SUMOi treatment (Fig. 5D; Supplemental Fig. S8G–I). SUMO inhibition also reduced telomere clustering upon targeting Rad52 to telomeres (Supplemental Fig. S8J). This effect was not seen for BLM or Rad51AP1. These results suggest that Rad52's effects on telomeres are uniquely connected to SUMOylation.

We asked whether Rad52 had the intrinsic ability to phase-separate and enrich SUMO or whether these effects resulted from Rad52's action at the telomeres. To this end, we used an established method to test Rad52's ability to phase-separate off telomeres in PML KO cells (Fig. 5E; Lackner et al. 2022; Zhang et al. 2018). This method uses a chemically induced protein dimerization approach similar to that described above, except that the protein is dimerized to a synthetic hexamer HOtag3 oligomer, which can increase interaction valence among the protein molecules. Live imaging showed diffuse signals in the nucleoplasm for both Rad52 and HOtag3 before dimerization (Fig. 5F; Supplemental Movie S9). After adding the dimerizer, condensates containing both Rad52 and HOtag3 were formed. In addition, the condensates coarsened over time through coalescence, suggesting liquid-like properties of the condensates. Since Rad52 in budding yeast has been reported to undergo phase separation to facilitate DNA repair (Oshidari et al. 2020), our observations with human Rad52 suggest that the ability of Rad52 to phase-separate is conserved.

Similar to Rad52, we found that the dimerization of Rad51AP1 and BLM to HOtag3 also led to condensate formation (Supplemental Fig. S9A,B; Supplemental Movies S10, S11). Phase diagram mapping indicated that BLM had a higher propensity to phase-separate than Rad52. In contrast, Rad51AP1 was less likely to phase-separate than Rad52 (Supplemental Fig. S9C). Interestingly, synthetic Rad52 condensates enrich SUMO2/3 and, to a lesser degree, SUMO1. This effect was not seen for BLM and Rad51AP1 condensates or a synthetic condensate formed by the arginine/glycine-rich (RGG) domain from the P-granule component LAF-1 protein (Fig. 5G,H; Supplemental Fig. S9D–F; Elbaum-Garfinkle et al. 2015; Schuster et al. 2018). Together, these results suggest that BLM, Rad52, and Rad51AP1 can all phase-separate, but only Rad52 has the intrinsic ability to enrich SUMO independent of PML.

### SUMO promotes Rad52 collaboration with BLM for telomere DNA synthesis

**Figure 6.**
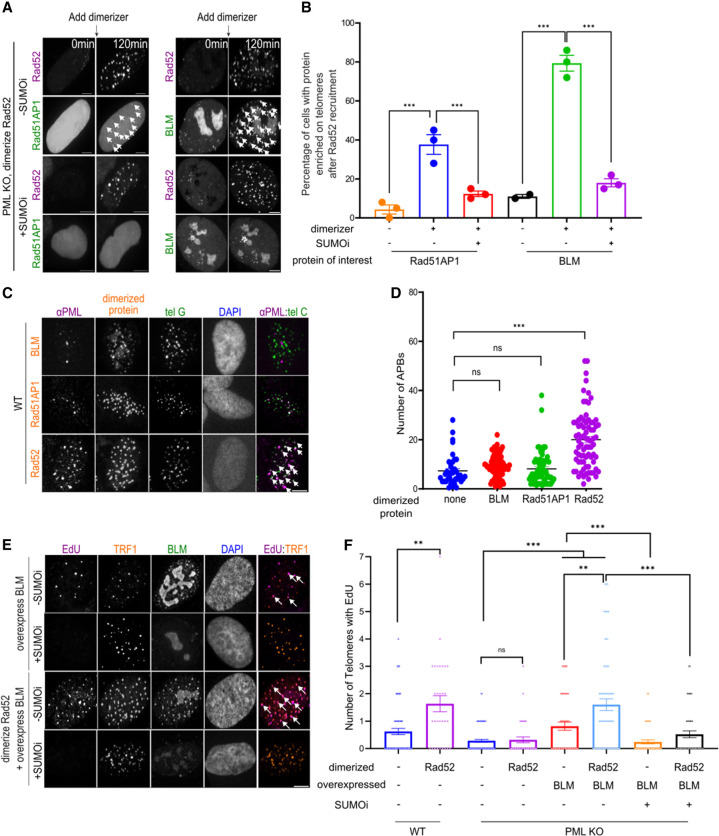
SUMO promotes Rad52 collaboration with BLM for telomere DNA synthesis. (*A*,*B*) Representative images (*A*) and quantification (*B*) of Rad51AP1 and BLM localization at telomeres after dimerizing mCh-eDHFR-Rad52 to 3xHalo-TRF1 in PML KO cells expressing GFP-Rad51AP1 or GFP-BLM with or without 1 μM SUMOi for 2 days. Each dot represents one experiment, three independent experiments, >32 cells per group. (*C*,*D*) Representative images (*C*) and quantification (*D*) of APB numbers in WT cells after dimerizing BLM/Rad51AP1/Rad52 to telomeres for 6 h. Each dot represents one cell, three independent experiments, >61 cells per group. (*E*,*F*) Representative images (*E*) and quantification (*F*) of EdU foci showing newly synthesized telomeric DNA after dimerizing Rad52 to telomeres in PML KO and WT U2OS cells with or without overexpressing BLM and treatment with 1 μM SUMOi for 2 days. White arrows indicate EdU signals at telomeres. Each dot represents one cell, three independent experiments, >30 cells per group. Scale bars, 5 μm.

To further explore the relationship between Rad52 and SUMO, we investigated whether Rad52 recruitment to telomeres could enrich other DNA repair factors in a SUMO-dependent manner. Live imaging showed enrichment of BLM and Rad51AP1 following recruitment of Rad52 to PML KO telomeres (Fig. 6A,B). The enrichment of both proteins, along with SUMO1 and SUMO2/3, was reduced after treatment with SUMOi (Fig. 6A,B). Conversely, recruitment of BLM and Rad51AP1 to telomeres in PML KO cells also enriched Rad52, and the enrichment was also reduced by SUMOi treatment (Supplemental Fig. S10A–C), although no SUMO enrichment was detected in these cases. We suspected this could be due to low levels of SUMO enrichment being difficult to visualize using microscopy. The ability of telomere targeted Rad52 to enrich BLM and Rad51AP1 was greater than Rad52 telomere enrichment upon BLM and Rad51AP1 targeting. Consistent with this difference, the recruitment of Rad52 to telomeres led to APB formation in PML-containing control cells, whereas the recruitment of Rad51AP1 and BLM did not (Fig. 6C,D).

Next, we examined the effects of telomere-targeting Rad52, BLM, and Rad51AP1 on telomere DNA synthesis. We found that targeting Rad52 and BLM, but not Rad51AP1, to telomeres was sufficient to induce telomere DNA synthesis in PML-containing cells (Fig. 6F; Supplemental Fig. S10D,E). However, in PML KO cells, BLM targeting to telomeres induced telomere DNA synthesis, and SUMO inhibition abolished BLM-induced telomere DNA synthesis, whereas these effects were not observed with Rad52 or Rad51AP1 targeting (Supplemental Fig. S10E,F). This finding confirmed the critical role of BLM in ALT telomere DNA synthesis (Min et al. 2019; Loe et al. 2020; Zhang et al. 2021) and further suggests that BLM function in ALT can be APB-independent while still requiring SUMO. Significantly, Rad52 telomere targeting enhanced telomere synthesis induced by overexpression of BLM in PML KO cells. This effect was dampened upon SUMOi treatment, indicating its dependence on SUMOylation (Fig. 6E,F). These data suggest that SUMO promotes Rad52 collaboration with BLM to support ALT telomeric DNA synthesis independent of PML and APBs.

### SUMO supports the viability of ALT cancer cells

**Figure 7.**
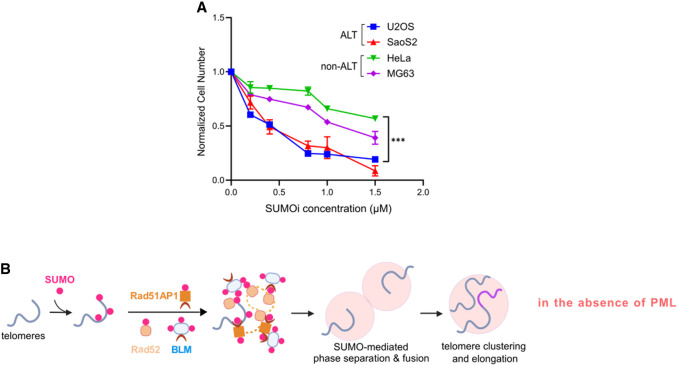
The SUMO pathway is important for ALT cells. (*A*) Normalized cell number after treatment with SUMOi at the indicated concentration for 10 days in U2OS, HeLa, Saos2, and MG63 cells. (*B*) Model showing that in the absence of PML, SUMOylation after DNA damage at ALT telomeres recruits DNA repair factors, such as Rad52, Rad51AP1, and BLM, and promotes their co-phase separation to form SUMO condensates for telomere clustering and elongation.

Our results suggest that SUMOylation plays an important role in enabling ALT through collaboration with DNA repair factors such as Rad52, in addition to its known function in APB formation. This dual role in ALT suggests that inhibition of SUMOylation may preferentially impair the growth of cells using the ALT pathway compared with cells using telomerase for telomere maintenance. To test this possibility, we investigated the effect of SUMO inhibition on cell survival by cell proliferation assay (Yadav et al. 2022; Cabrini et al. 2023). We applied 0–2 μM SUMOi to the ALT-positive cell lines U2OS and Saos2 (osteosarcoma-derived) and to the non-ALT cancer cell lines HeLa and MG63 for 10 days. As SUMOi concentration increased, U2OS and SaoS2 cell numbers decreased dramatically. In contrast, HeLa and MG63 cell numbers were less affected (Fig. 7A). This result suggests that SUMOylation inhibition preferentially kills ALT cells over non-ALT cells.

## Discussion

Previous studies have established the important role of SUMO in ALT in promoting APB formation. Here, we address whether SUMO contributes to ALT in the absence of PML and APBs. We provide multiple lines of evidence to support the intrinsic ability of SUMOylation to promote multiple ALT features in PML KO cells. We show that this ability is mediated by SUMO–SIM interactions. We have identified several DNA repair proteins involved in ALT that can mediate SUMO-based contributions to ALT features independent of PML (Fig. 7B). We further unveiled a unique ability of Rad52 to mediate the enrichment of SUMO at telomeres and to enable SUMO-based telomere clustering and telomeric DNA synthesis. Collectively, our work defines the important roles of SUMOylation and SUMO–SIM interactions in promoting ALT pathways independent of PML.

The utilization of a SUMO inhibitor and a chemically induced protein–protein dimerization method have enabled us to examine the roles of SUMOylation and telomere targeting of SUMO isoforms, SIM, and DNA repair proteins. We investigated endogenous ALT features and those induced by telomere breaks. Using both live-cell imaging and immunofluorescence imaging, we systematically examined the telomere localization of these proteins and studied telomere clustering and telomeric DNA synthesis. Our data suggest that SUMOylation at ALT telomeres can directly recruit DNA repair proteins via SUMO–SIM interactions and drive telomere clustering and telomere DNA synthesis in the absence of PML and APBs (Fig. 7B). The proteins enriched via SUMO targeting include RPA and BLM, which are known to be important for ALT, as well as Rad52 and Rad51AP1, which control two different ALT pathways, suggesting that SUMO can mediate both branches of ALT. We thus conclude that SUMOylation is a fundamental requirement for ALT. The influence of SUMOylation is not limited to APBs, since it can promote ALT in PML-null cells by collaborating with DNA repair factors. These data suggest that targeting the SUMO pathway using SUMOi can efficiently abolish ALT, thus providing a possible approach for ALT cancer therapy. Indeed, we found that SUMOi treatment preferentially impairs the growth of ALT cells.

Among the HR proteins (Rad51, Rad51AP1, and Rad52) implicated in competing parallel pathways in ALT, Rad51 was not recruited using SUMO, whereas Rad51AP1 and RAD52 were. In addition, Rad52 was the only HR protein that, when targeted to telomeres, could enrich SUMO at telomeres and enhance telomere clustering and telomeric DNA synthesis. The unique interplay between SUMO and RAD52 suggests that SUMO may have a role in determining which ALT pathway is used following the formation of DNA breaks at telomeres. The biochemical nature of Rad52 that renders its unique connection with SUMO in the ALT pathway remains to be determined. We speculate that these features may be related to the ability of Rad52 to interact with the SUMO E2 enzyme UBC9 (Dou et al. 2010, 2011). This interaction has not been reported for other proteins involved in ALT. Future work to test this hypothesis and other possible mechanisms can further clarify the roles of Rad52 in ALT.

Previous studies have shown that SUMO mediates PML phase separation to form APBs (Zhang et al. 2020). We now report signatures of phase separation after recruiting SUMO in PML KO cells. We speculate that this effect can be linked to the intrinsic ability of the telomere binding proteins and DNA repair factors to phase-separate. Indeed, TRF1/TRF2 and RPA have been demonstrated to undergo phase separation (Jack et al. 2022; Spegg et al. 2023). Here, we show that Rad52, BLM, and Rad51AP1 can undergo different degrees of phase separation. Since SUMO has an established role in acting as a molecular glue to promote the coenrichment of repair factors at the DNA damage site (Psakhye and Jentsch 2012), we suggest a model in which SUMO mediates co-phase separation of DNA repair factors and telomere binding proteins at ALT telomeres (Fig. 7B). We suspect that SUMO-mediated co-phase separation of DNA repair factors may also be used at nontelomere DNA damage sites to enrich multiple DNA repair proteins and DNA substrates to achieve better repair efficiency. Future experiments to test these ideas will further clarify how SUMO collaborates with various DNA repair factors in genome protection.

It is worth noting that the examined DNA repair proteins have a lower propensity to phase-separate than PML because the expression of these proteins alone in cells did not lead to the formation of condensates. Therefore, whereas SUMO can promote ALT in the absence of PML, PML can enhance SUMO-mediated phase separation of repair factors at ALT telomeres. The collaboration of SUMO and PML thus can lead to more efficient telomere clustering and telomeric DNA synthesis. Indeed, PML KO cells lose telomere heterogeneity and exhibit telomere shortening over time, suggesting low efficiency in maintaining telomere length without PML (Loe et al. 2020). In conclusion, our work unveils an important role of SUMO in promoting condensate formation for ALT telomere maintenance, either through collaboration with DNA repair factors or with PML.

## Materials and methods

### Cell culture

All WT experiments were performed with U2OS cells. PML KO cells were gifts from Dr. Eros Lazzerini Denchi. U2OS cells with endogenous PML tagged with Clover (U2OS Clover-PML) were previously described (Pinder et al. 2015). Saos2 and MG63 were gifts from Dr. Samantha Pattenden. HeLa 1.3 cells were a gift from Dr. Roger A. Greenberg. Cells were cultured in growth medium (Dulbecco's modified Eagle's medium with 10% FBS and 1% penicillin/streptomycin) at 37°C in a humidified atmosphere with 5% CO_2_.

### Plasmids

The plasmid for inducing DNA damage at telomeres (mCherry-TRF1-FokI) was previously published (Cho et al. 2014). 3xHalo-GFP-TRF1 was previously published (Zhang et al. 2020). SUMO1/2/3 (or SUMO mutant) for mCherry-eDHFR-SUMO is from plasmids gifted by Karsten Rippe (Chung et al. 2011). SIM and SUMO mutants for mCherry-eDHFR-SIM/SUMO are from plasmids gifted by Michael Rosen, where the SIM-interacting mutants of SUMO were generated by mutating the FKIK (SUMO3) or FKVK (SUMO1) to FAAA, nonconjugatable SUMO modules were generated by mutating the C-terminal di-Gly motif to di-Val, and the mutant of SIM defective in SUMO binding was generated by mutating the VIDL sequence into VADA (Banani et al. 2016). The vector containing mCherry-eDHFR is from our published plasmid Mad1-mCherry-eDHFR (Zhang et al. 2017). NLS was cloned in 3xHalo-GFP-HOTag3 (Lackner et al. 2022). RGG-mCherry-RGG-eDHFR was previously published (Lackner et al. 2022). GFP-BLM is from Addgene plasmid 80070. GFP-Rad51AP1 and mutants were gifted by R.J.O. and introduced into target plasmids through in-fusion cloning (Takara Bio 638948). All the target plasmids in this study are derived from a plasmid that contains a CAG promoter for constitutive expression that was obtained from E.V. Makeyev (Khandelia et al. 2011).

### Cell treatment with chemicals

SUMO inhibitor ML-792 (Selleck Chemicals HY-108702), used to inhibit SUMOylation, was added to cells at 1 μM for 2 days. For triggering telomere DNA damage in FokI cells, 4-hydroxytamoxifen (4-OHT; Sigma-Aldrich H7904) was added to cells at 1 μM working concentration for the indicated time.

### Synchronizing cells to G2

To synchronize cells to G2, cells were first treated with 2 mM thymidine (Sigma-Aldrich T1895) for 21 h, released into fresh medium for 4 h, and then treated with 15 μM CDK1 inhibitor (RO-3306; cSigma-Aldrich SML0569) for 12 h.

### Quantitative real-time PCR

Total RNAs were isolated using RNA miniprep (Zymo NC1047980), and 1 μg of RNA was used for reverse transcription using the iScript cDNA synthesis kit (Bio-Rad 1708891). Quantitative PCR reactions were done with equal amounts of cDNA using a DyNAmo SYBR Green qPCR kit (Thermo Fisher Scientific F410). Signals were detected by QuantStudio 3 (Thermo Fisher A28567). GAPDH was used as the internal control. The primer was used as described (Silva et al. 2019).

### siRNA transfection

siRNAs for PML (Dharmacon J-006547-05-0002) and POLD3 (Thermo Fisher 4390824) were purchased commercially. Cells were transfected with 100 nM siRNA and RNAiMax (Thermo Fisher 13778075) diluted in OptiMEM (Life Technologies 31-985-070). The transfection medium was replaced with culture media 8 h later, the transfection was repeated on day 2, and cells were collected and imaged at 48 h after the second round of transfection.

### Protein dimerization with chemical dimerizers

Design, synthesis, and storage of the dimerizer TMP (trimethoprim)–fluorobenzamide–Halo ligand (TFH) were reported previously (Lackner et al. 2022). Dimerization to telomeres was performed as previously described (Xu et al. 2022). Briefly, TFH was added directly to the growth medium to a final working concentration of 100 nM. Cells were incubated with the dimerizer-containing medium for the indicated times, followed by immunofluorescence (IF) or fluorescence in situ hybridization (FISH). For live imaging with protein dimerization, the dimerizers are first diluted to 200 nM in growth medium and then further added to cell chambers to the working concentration after the first round of imaging.

### IF and telomere DNA FISH

IF and FISH were performed following a previously published protocol (Zhao et al. 2021). Cells were fixed in 4% formaldehyde for 10 min at room temperature, followed by permeabilization in 0.5% Triton X-100 for 10 min. Cells were incubated with primary antibody in a humidified chamber overnight at 4°C and then with secondary antibody for 1 h at room temperature before washing and mounting. Primary antibodies were anti-SUMO1 (1:200 dilution; Abcam ab32058), anti-SUMO2/3 (1:200 dilution; Cytoskeleton Asm23), anti-PML (1:50 dilution; Santa Cruz Biotechnology sc966), anti-Rad51 (1:200 dilution; Abcam ab213), and anti-RPA (1:100 dilution; Novus NB600-565). For IF-FISH, coverslips were first stained with primary and secondary antibodies and then fixed again in 4% formaldehyde for 10 min at room temperature. Coverslips were then dehydrated in an ethanol series (70%, 80%, and 90% for 2 min each) and incubated with a 488-tel G or Cy5-tel G PNA probe (Panagene F1007 and F1008) for 5 min at 75°C and then overnight in a humidified chamber at room temperature. Coverslips were then washed and mounted for imaging.

### Telomere DNA synthesis detection by EdU

Following transfection, cells were pulsed with 10 μM EdU along with protein dimerization or DNA damage induction for 6 h before harvest. Cells on glass coverslips were washed twice in PBS and fixed with 4% paraformaldehyde (PFA) for 10 min. Cells were permeabilized with 0.3% (v/v) Triton X-100 for 5 min. The Click-IT Plus EdU cell proliferation kit with Alexa flour 488 or 647 (Invitrogen C10633 and C10635) was applied to cells for 30 min to detect EdU.

### Cell imaging and image processing

Image acquisition was performed as previously described (Xu et al. 2024). For live imaging, cells were seeded onto 22 mm × 22 mm glass coverslips coated with poly-D-lysine (Sigma-Aldrich P1024). When ready for imaging, coverslips were mounted in magnetic chambers (LCI Chamlide CM-S22-1), with cells maintained in a normal medium supplemented with 10% FBS and 1% penicillin/streptomycin at 37°C on a heated stage in an environmental chamber (Tokai Hit Co., Ltd.). Images were acquired with a microscope (Eclipse Ti2) with a 100× 1.4 NA objective, a 16 XY Piezo-Z stage (Nikon Instruments, Inc.), a spinning disk (Yokogawa), an electron multiplier charge-coupled device camera (IXON-L-897), and a laser merge module that was equipped with 488, 561, 594, and 630 nm lasers controlled by NIS-Elements Advanced Research (AR). For both fixed cells and live imaging, images were taken with 0.5 μm spacing between *Z* slices for a total of 8 μm. For movies, images were taken at 5 min intervals for up to 3 h.

Images were processed and analyzed using NIS-Elements AR (Nikon). Maximum projections were created from *z*-stacks, and thresholds were applied to the resulting 2D images to segment and identify telomere/SUMO foci as binaries. For colocalization quantification of two fluorescent labels, images were analyzed using binary operations in NIS-Elements AR. Colocalized foci were counted if the objects from different layers contained overlapping pixels.

### Crystal violet staining for cell number

Around 2000 cells were seeded in 6 well plates. The next day, cells were treated with various concentrations of SUMOi for 10 days for colony formation. Media was aspirated off and crystal violet was added until it filled the wells. After shaking for 5 min, the crystal violet was removed, water was added, and the plate was swirled gently to wash the remaining stain off. Next, the plate was moved to a Tecan Spark plate reader to measure OD at 590 nm.

### C-circle assay

Genomic DNA was purified, digested with AluI and MboI, and cleaned by phenol-chloroform extraction and precipitation. DNA was diluted in ultraclean water, and concentrations were measured to the indicated quantity (30, 15, and 7.5 ng) using a NanoDrop (Thermo Fisher). Samples (10 μL) were combined with 10 μL of 0.2 mg/mL BSA (NEB); 0.1% Tween; 0.2 mM each dATP, dGTP, and dTTP; and 1× Φ29 buffer (NEB) in the presence or absence of 7.5 U of ΦDNA polymerase (NEB). Samples were incubated for 8 h at 30°C and then for 20 min at 65°C. Reaction products were diluted to 100 μL with 2× saline-sodium citrate (SSC) buffer and dot-blotted onto a 2× SSC-soaked nylon membrane. DNA was ultraviolet (UV)-cross-linked onto the membrane and hybridized with a biotin-labeled (CCCTAA)_4_ oligonucleotide probe (PNA Bio F2001) to detect C-circle amplification products. The HRP signal was visualized with a chemiluminescent nucleic acid detection module kit (Thermo 89880) per the manufacturer's instructions.

### Western blotting

Cells were harvested with trypsin, quickly washed in PBS, and directly lysed in 4× NuPage LDS sample buffer (Thermo NP0007). The resulting whole-cell lysates were analyzed by Western blotting with the following primary antibodies: PML (Santa Cruz Biotechnology sc966), POLD3 (Abnova H00010714-M01), and β-Actin (Abcam ab8226). The HRP signal was visualized with Super Signal ECL substrate (Pierce 34095) per the manufacturer's instructions.

### Statistical methods

All error bars represent means ± SEM. Statistical analyses were performed using Prism 10.0 (GraphPad Software). Two-tailed unpaired *t*-tests were used for all tests. Statistical significance is indicated in the figures as follows: not significant (*P* > 0.05; N.S.), *P* < 0.05 (*), *P* < 0.01 (**), and *P* < 0.001 (***).

### Data availability

All data needed to evaluate the conclusions in this study are presented here and in the Supplemental Material.

## Supplementary Material

Supplement 1

Supplement 2

Supplement 3

Supplement 4

Supplement 5

Supplement 6

Supplement 7

Supplement 8

Supplement 9

Supplement 10

Supplement 11

Supplement 12
