# Supplementary material for: SUMO promotes DNA repair protein collaboration to support alternative telomere lengthening in the absence of PML
Source: Genes Dev. 2024 Jul 1;38(13-14):614–30. doi: 10.1101/gad.351667.124 (PMC11368244; doi:10.1101/gad.351667.124)
Supplement: Supplement 1 [file Supplemental_Data.pdf]

Supplementary Materials for

# SUMO Promotes DNA Repair Protein Collaboration to Support Alterative Telomere Lengthening in the Absence of PML

Rongwei Zhao *et al.*

* Corresponding author: huaiyinz@andrew.cmu.edu

**This file includes:**

Supplementary Figures 1 to 10; Supplementary Movies 1 to 11

**Movie 1** Inducing DNA damage at PML KO telomeres. Movie for Fig. 1D. 4-OHT was added to cells after the first time point to induce damage. The box shows a telomere fusion event. Scale bars, 5 µm.

**Movie 2** Dimerizing SUMO3 to PML KO telomeres. Movie for Fig. 3B. Left: Composite of SUMO3 (magenta) and TRF1 (green), middle: mCh-eDHFR-SUMO3, right: 3xHalo-GFP-TRF1. The dimerizer was added to cells after the first time point to induce dimerization. The box shows a telomere fusion event. Scale bars, 5 µm.

**Movie 3** Dimerizing SUMO1 to PML KO telomeres. Movie for Fig. S4A. Left: Composite of SUMO1 (magenta) and TRF1 (green), middle: mCh-eDHFR-SUMO1, right: 3xHalo-GFP-TRF1. The dimerizer was added to cells after the first time point to induce dimerization. The box shows a telomere fusion event. Scale bars, 5 µm.

**Movie 4** Dimerizing SUMO2 to PML KO telomeres. Movie for Fig. S4A. Left: Composite of SUMO2 (magenta) and TRF1 (green), middle: mCh-eDHFR-SUMO2, right: 3xHalo-GFP-TRF1. The dimerizer was added to cells after the first time point to induce dimerization. The box shows a telomere fusion event. Scale bars, 5 µm.

**Movie 5** Dimerizing SUMO3 SIM interacting mutant to PML KO telomeres. Movie for Fig. 3B. Left: Composite of SUMO3 mutant (magenta) and TRF1 (green), middle: mCh-eDHFR-SUMO3m, right: 3xHalo-GFP-TRF1. The dimerizer was added to cells after the first time point to induce dimerization. Scale bars, 5 µm.

**Movie 6** Dimerizing Rad52 to PML KO telomeres. Movie for Fig. 5B. Left: Composite of Rad52 (magenta) and TRF1 (green), middle: mCh-eDHFR-Rad52, right: 3xHalo-GFP-TRF1. The dimerizer was added to cells after the first time point to induce dimerization. The box shows a telomere fusion event. Scale bars, 5 µm.

**Movie 7** Dimerizing BLM to PML KO telomeres. Movie for Fig. S8A. Left: Composite of BLM (magenta) and TRF1 (green), middle: mCh-eDHFR-BLM, right: 3xHalo-GFP-TRF1. The dimerizer was added to cells after the first time point to induce dimerization. The box shows a telomere fusion event. Scale bars, 5 µm.

**Movie 8** Dimerizing Rad51AP1 to PML KO telomeres. Movie for Fig. S8C. Left: Composite of Rad51AP1 (magenta) and TRF1 (green), middle: mCh-eDHFR-Rad51AP1, right: 3xHalo-GFP-TRF1. The dimerizer was added to cells after the first time point to induce dimerization. The box shows a telomere fusion event. Scale bars, 5 μm.

**Movie 9** Dimerizing Rad52 to HOtag3 in PML KO cells. Movie for Fig. 5F. Left: Composite of Rad52 (magenta) and HOtag3 (green), middle: mCh-eDHFR-Rad52, right: 3xHalo-GFP-HOtag3. The dimerizer was added to cells after the first time point to induce dimerization. The box shows a droplet fusion event. Scale bars, 5 μm.

**Movie 10** Dimerizing BLM to HOtag3 in PML KO cells. Movie for Fig. S9A. Left: Composite of BLM (magenta) and HOtag3 (green), middle: mCh-eDHFR-BLM, right: 3xHalo-GFP-HOtag3. The dimerizer was added to cells after the first time point to induce dimerization. The box shows a droplet fusion event. Scale bars, 5 μm.

**Movie 11** Dimerizing Rad51AP1 to HOtag3 in PML KO cells. Movie for Fig. S9B. Left: Composite of Rad51AP1 (magenta) and HOtag3 (green), middle: mCh-eDHFR-Rad51AP1, right: 3xHalo-GFP-HOtag3. The dimerizer was added to cells after the first time point to induce dimerization. Scale bars, 5 μm.

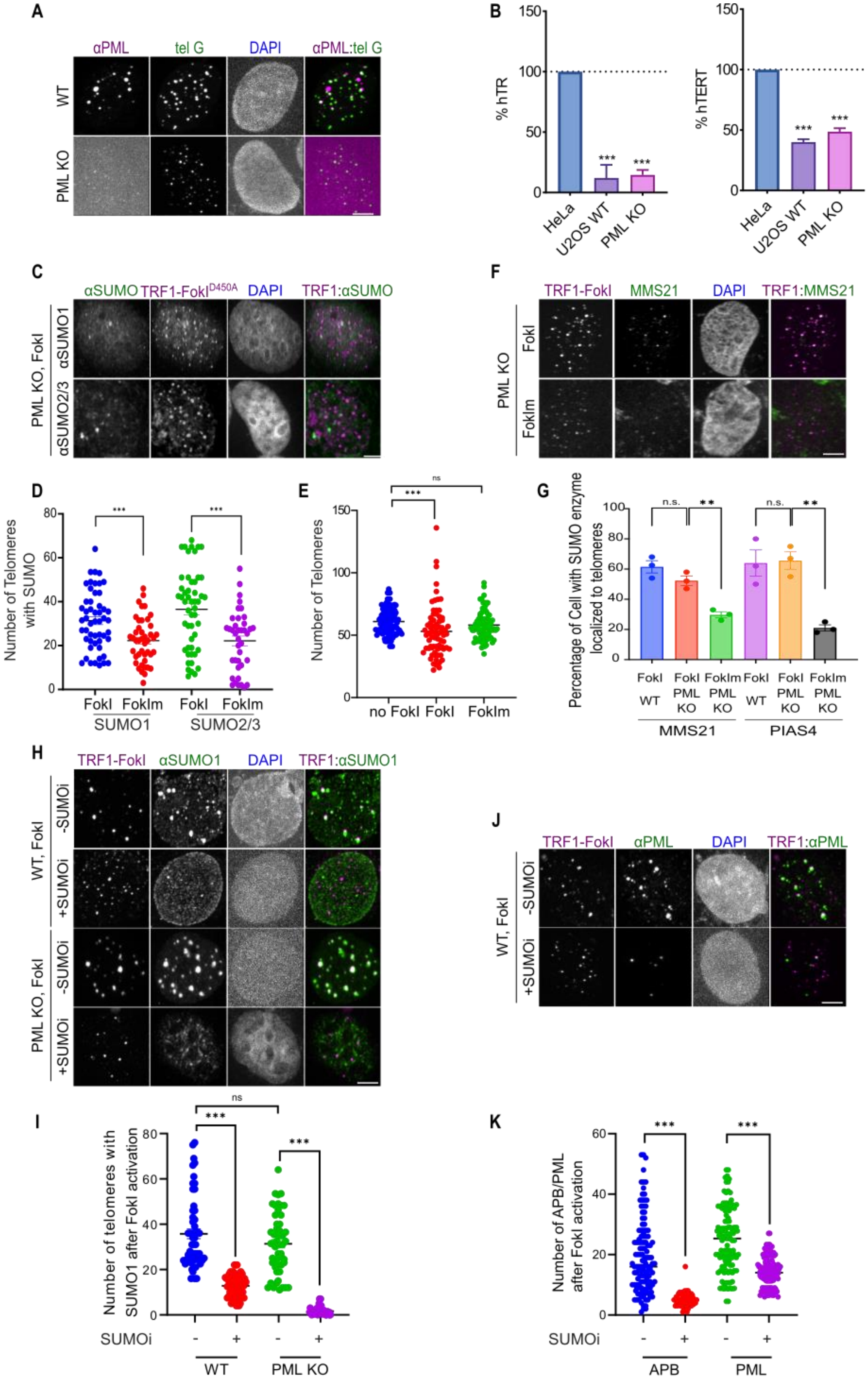
A
αPML
tel G
DAPI
αPML:tel G
WT
PML KO
B
% hTR
% hTERT
HeLa
U2OS WT
PML KO
***
C
αSUMO
TRF1-FokI D450A
DAPI
TRF1:αSUMO
PML KO, FokI
αSUMO1
αSUMO2/3
F
TRF1-FokI
MMS21
DAPI
TRF1:MMS21
PML KO
FokI
FokIm
D
Number of Telomeres with SUMO
FokI
FokIm
SUMO1
SUMO2/3
E
Number of Telomeres
ns
no FokI
FokI
FokIm
G
Percentage of Cell with SUMO enzyme localized to telomeres
n.s.
**
FokI WT
FokI PML KO
FokIm PML KO
MMS21
PIAS4
H
TRF1-FokI
αSUMO1
DAPI
TRF1:αSUMO1
WT, FokI
PML KO, FokI
-SUMOi
+SUMOi
J
TRF1-FokI
αPML
DAPI
TRF1:αPML
WT, FokI
-SUMOi
+SUMOi
I
Number of telomeres with SUMO1 after FokI activation
SUMOi
WT
PML KO
K
Number of APB/PML after FokI activation
SUMOi
APB
PML

**Fig. S1. ALT features in WT and PML KO U2OS cells after FokI activation with or without SUMOi. (A)** Representative images of PML staining in WT and PML KO cells. **(B)** hTR and hTERT expression levels in HeLa, U2OS WT, and PML KO cells. **(C)** Representative images of SUMO1/2/3 localization at telomeres in PML KO expressing TRF1-FokI-D450A after adding 4-Hydroxyestradiol (4-OHT) for 6 hours. **(D)** Quantification of SUMO1/2/3 localization at telomeres and **(E)** telomere numbers in PML KO with or without expressing TRF1-FokI-WT and TRF1-FokI-D450A to induce DNA damage for 6 hours. Each dot represents one cell, three independent experiments, more than 56 cells per group. **(F)** Representative images and **(G)** quantification of MMS21 and PIAS4 localization at telomeres in U2OS WT and PML KO cells expressing mCh-TRF1-FokI and FokI enzymatic dead mutant TRF1-FokI-D450A with treatment of 4-Hydroxyestradiol (4-OHT) for 6 hours. Each dot represents one experiment, three independent experiments, more than 41 cells per group. **(H)** Representative images and **(I)** Quantification of SUMO1 localization at telomeres in WT and PML KO cells with or without 1 μM SUMOi under 6-hour FokI-induced DNA damage. **(J)** Representative images and **(K)** quantification of APB and PML body numbers in WT under FokI-induced DNA damage for 6 hours with or without SUMOi. Each dot represents one cell, three independent experiments, more than 63 cells per group. Scale bars, 5 μm.

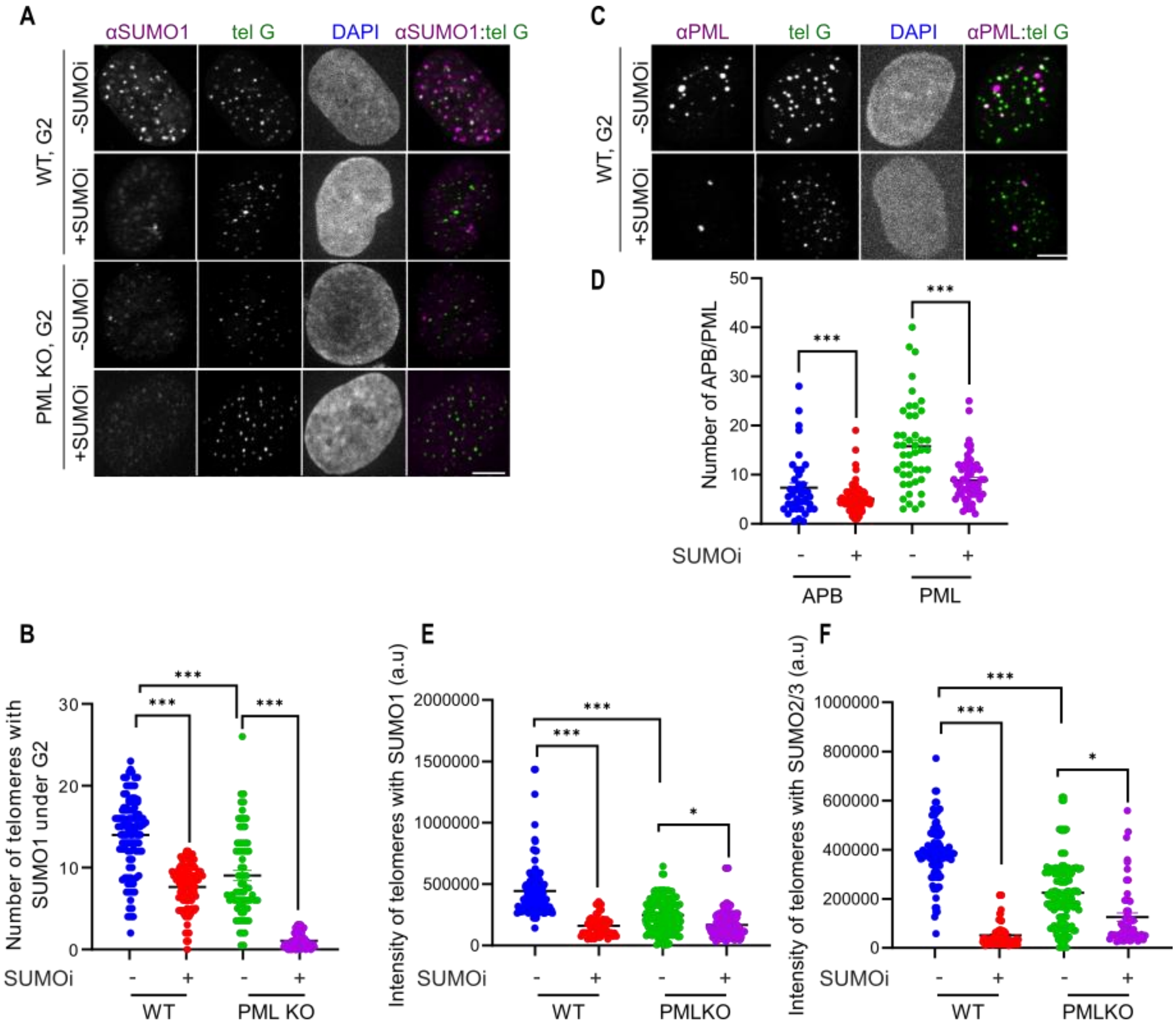


**Fig. S2. ALT features in G2 arrested WT and PML KO cells with or without SUMOi treatment.** **(A)** Representative images and **(B)** quantification of SUMO1 localization at telomeres in G2 arrested-WT and PML KO cells with or without 1 μM SUMOi after 2 days. Each dot represents one cell, three independent experiments, more than 70 cells per group. **(C)** Representative images and **(D)** quantification of APB and PML body number in G2 arrested-WT treating with or without 1 μM SUMOi after 2 days. Each dot represents one cell, three independent experiments, more than 62 cells per group. **(E, F)** Quantification of the intensity of telomeres with SUMO1 or SUMO2/3 localized in WT and PML KO U2OS cells with or without SUMOi. Each dot represents one cell, three independent experiments, more than 90 cells per group. Scale bars, 5 μm.

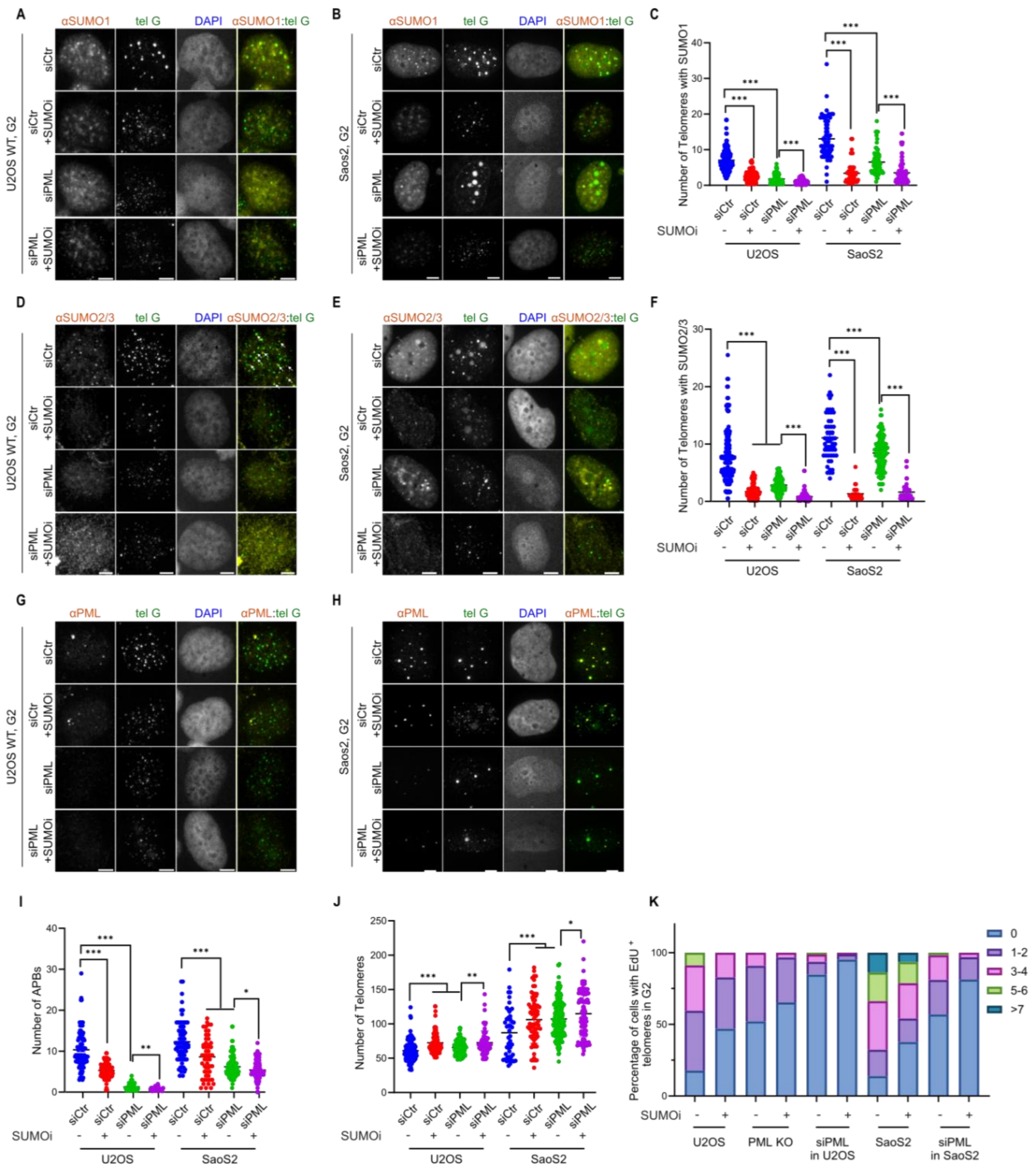


**Fig. S3. ALT features in G2 arrested-U2OS and Saos2 cells with/without SUMOi treatment. (A-B)** Representative images and **(C)** quantification of SUMO1 localization at telomeres in U2OS and Saos2 cells after transfecting control siRNA or siPML for 2 days, with or without treatment with 1 µM SUMOi for 2 days. Each dot represents one cell, three independent experiments, more than 82 cells per group. **(D, E)** Representative images and **(F)** quantification

of SUMO2/3 localization at telomeres in U2OS and Saos2 cells after transfecting control siRNA or siPML for 2 days, with or without treating 1 μM SUMOi for 2 days. White arrows indicate SUMO localization at telomeres. Each dot represents one cell, three independent experiments, more than 55 cells per group. **(G, H)** Representative images and **(I)** quantification of APB numbers in U2OS and Saos2 cells after transfecting control siRNA or siPML for 2 days, with or without treating with 1 μM SUMOi for 2 days. **(J)** Number of telomeres in U2OS and Saos2 after transfecting control siRNA or siPML for 2 days, with or without treating 1 μM SUMOi for 2 days. Each dot represents one cell, three independent experiments, more than 63 cells per group. **(K)** Quantification for cells with different numbers of EdU positive telomeres (0, 1-2, 3-4, 5-6 and ⩾ 7) in G2 synchronized U2OS PML KO cells, U2OS and Saos2 cells treated with control or PML siRNAs, with or without SUMOi. Three independent experiments, more than 37 cells in each group. Scale bars, 5 μm.

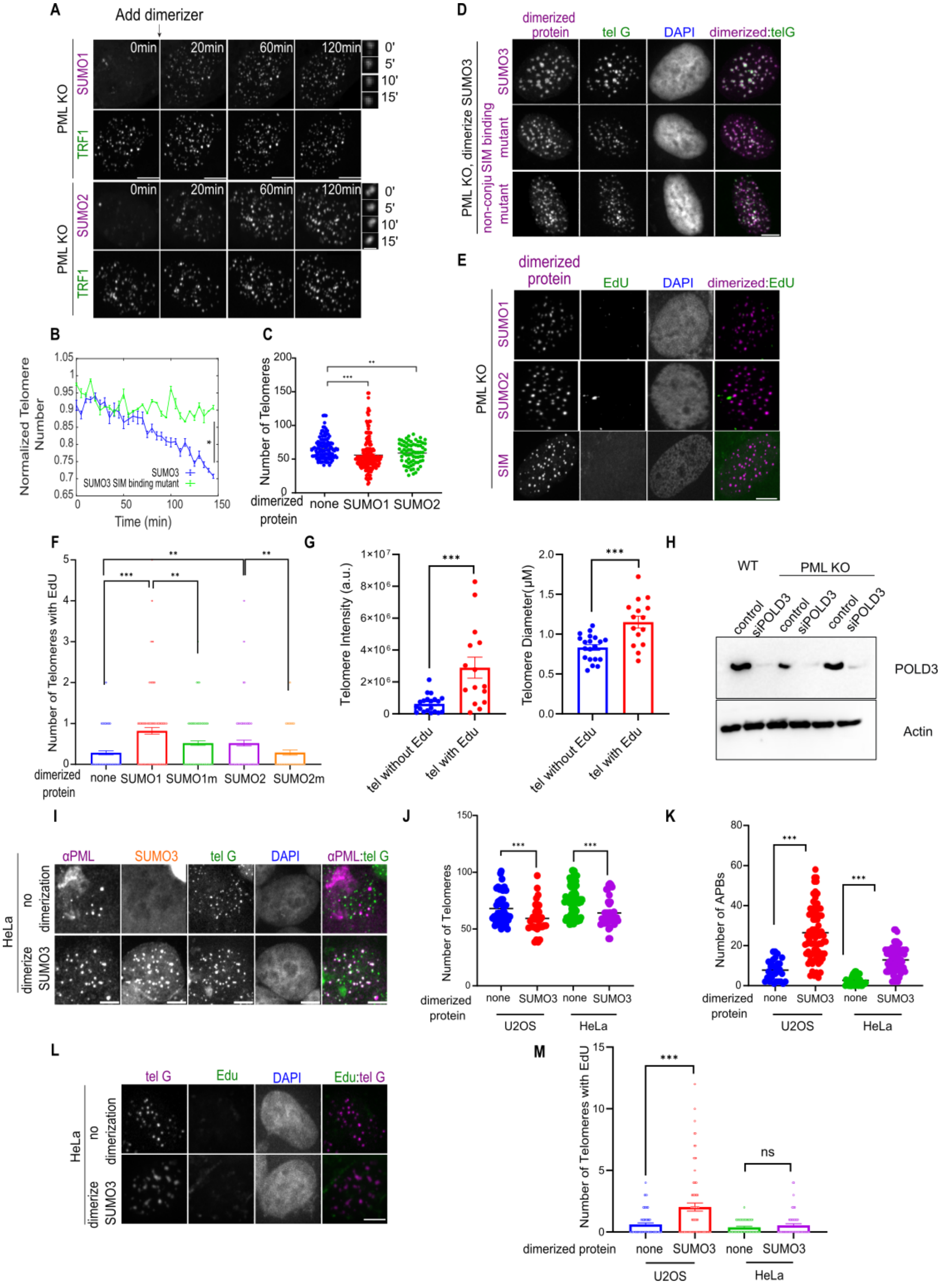


**Fig. S4. ALT features after dimerizing SUMO to telomeres in PML KO U2OS cells and Hela cells. (A)** Representative images of PML KO cells after dimerizing mCh-eDHFR-

SUMO1/SUMO2 to 3xHalo-GFP-TRF1 after the first time point. **(B)** Telomere number per cell over time from live images after dimerizing SUMO3 or SUMO3 mutant that cannot interact with SIM. Telomere numbers are normalized by the number at the first time point for each cell (more than 22 cells per group, three independent experiments, two-tailed unpaired *t-test)*. **(C)** Telomere number after dimerizing mCh-eDHFR-SUMO1/2 to 3xHalo-TRF1 in PML KO cells (more than 50 cells per group, three independent experiments, two-tailed unpaired-test). **(D)** Representative images of telomere DNA FISH after dimerizing SUMO3 or SUMO3 mutant to PML KO telomeres for 6 hours. **(E)** Representative images and **(F)** quantification of EdU assay showing newly synthesized telomeric DNA without dimerizing any protein or dimerizing SUMO1/2 and their SIM interaction mutants to telomeres for 6 hours in PML KO cells. Each dot represents one cell, three independent experiments, more than 66 cells in each group. **(G)** Quantification of telomere intensity and telomere size with or without EdU foci after dimerizing SUMO3 to PML KO telomeres. Each dot represents one cell, three independent experiments, more than 30 cells in each group. **(H)** Western blot of POLD3 after transfecting control siRNA or siPOLD2 in WT and PML KO U2OS cells. **(I)** Representative images and **(J, K)** quantification of telomere and APB numbers in U2OS and HeLa cells with or without dimerizing SUMO3 to telomeres for 6 hours. Each dot represents one cell, three independent experiments, more than 41 cells in each group. **(L)** Representative images and **(M)** quantification of EdU at telomeres in U2OS and HeLa cells with or without dimerizing SUMO3 to telomeres for 6 hours. Each dot represents one cell, three independent experiments, more than 44 cells in each group. Scale bars, 5 μm or 1 μm for the zoomed-in images.

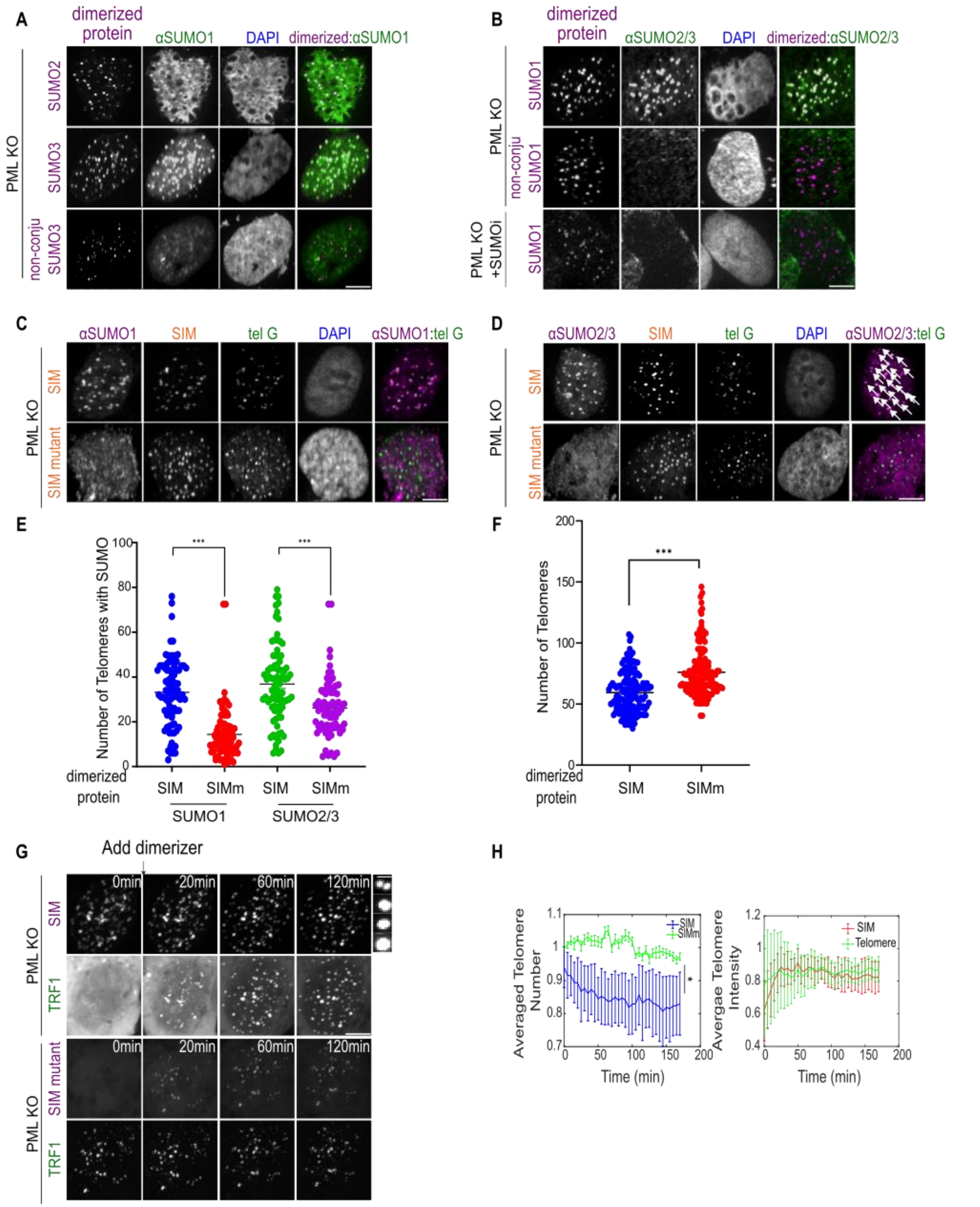


**Fig. S5. ALT phenotypes after dimerizing SIM and SUMO mutants to telomeres in PML KO U2OS. (A)** Immunofluorescence images of SUMO1 in PML KO cells dimerizing mCh-eDHFR-SUMO2/3, non-conjugatable SUMO3 to telomeres for 6 hours. **(B)** Immunofluorescence images of SUMO2/3 in PML KO cells after dimerizing mCh-eDHFR-SUMO1 and non-conjugatable

SUMO1 to telomeres for 6 hours, with/without SUMOi. **(C, D)** Immunofluorescent images and **(E)** quantification of SUMO1/2/3 localization at telomeres and **(F)** telomere numbers in PML KO after dimerizing SIM or SIM mutant to telomeres for 6 hours. Each dot represents one cell, three independent experiments, more than 77 cells in each group. **(G)** Representative images of PML KO cells after dimerizing mCh-eDHFR-SIM, or SIM mutant to 3xHalo-GFP-TRF1 at indicated time points. Zoomed-in images show a fusion event of TRF1 foci. **(H)** Telomere number, telomere sum intensity per cell after dimerizing SIM or SIM mutant to PML KO telomeres (more than 21 cells per group, three independent experiments, two-tailed unpaired t-test). Scale bars, 5 μm or 1 μm for zoomed-in images.

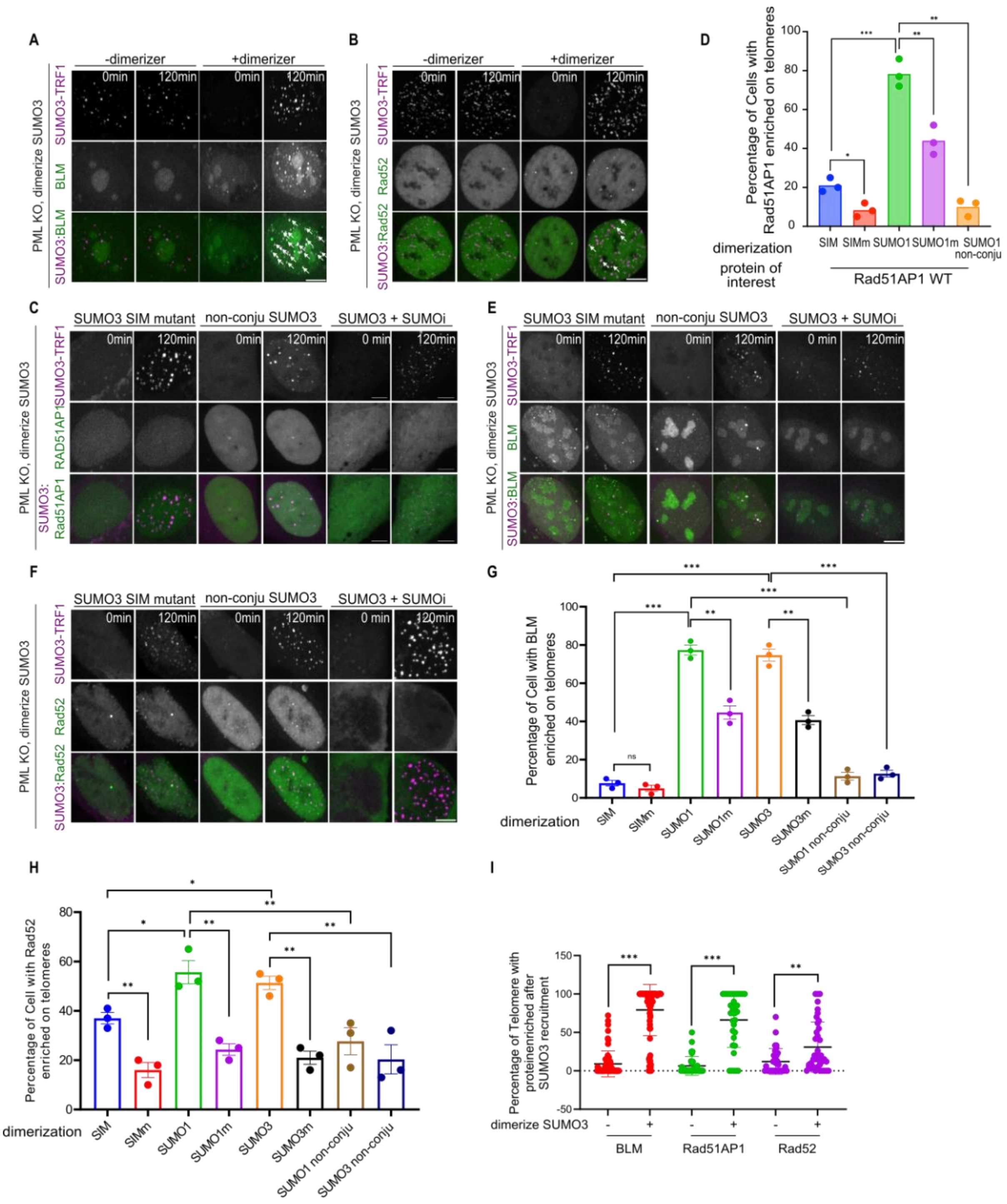


**Fig. S6. Localization of DNA repair factors to telomeres after dimerizing SUMO to PML KO telomeres. (A)** Representative images of BLM and **(B)** Rad52 localization at telomeres after dimerizing SUMO3 to telomeres in PML KO cells. White arrows indicate BLM or Rad52 localization at telomeres. **(C, D)** Representative images and quantification of the localization of

Rad51AP1 and mutants at telomeres after dimerizing mCh-eDHFR-SUMO3/SIM-binding mutant/non-conjugable mutant to 3xHalo-TRF1, with/without SUMOi in PML KO cells expressing GFP-Rad51AP1. Each dot represents one independent experiment, three independent experiments, more than 26 cells in each group. **(E, G)** Representative images and quantification of the localization of BLM at telomeres after dimerizing mCh-eDHFR-SUMO3/SIM-binding mutant/non-conjugable mutant to 3xHalo-TRF1, with/without SUMOi in PML KO cells expressing GFP-BLM. Each dot represents one experiment, three independent experiments, more than 34 cells in each group. **(F, H)** Representative images and quantification of the localization of Rad52 at telomeres after dimerizing mCh-eDHFR-SUMO3/SIM-binding mutant/non-conjugable mutant to 3xHalo-TRF1, with/without SUMOi in PML KO cells expressing GFP-Rad52. Each dot represents one independent experiment, three independent experiments, 32 cells in each group. **(I)** Percentage of telomeres per cell with BLM, Rad51AP1, or Rad52 enriched after recruiting SUMO3 to PML KO telomeres. Each dot represents one cell, three independent experiments, more than 39 cells in each group. Scale bars, 5 μm.

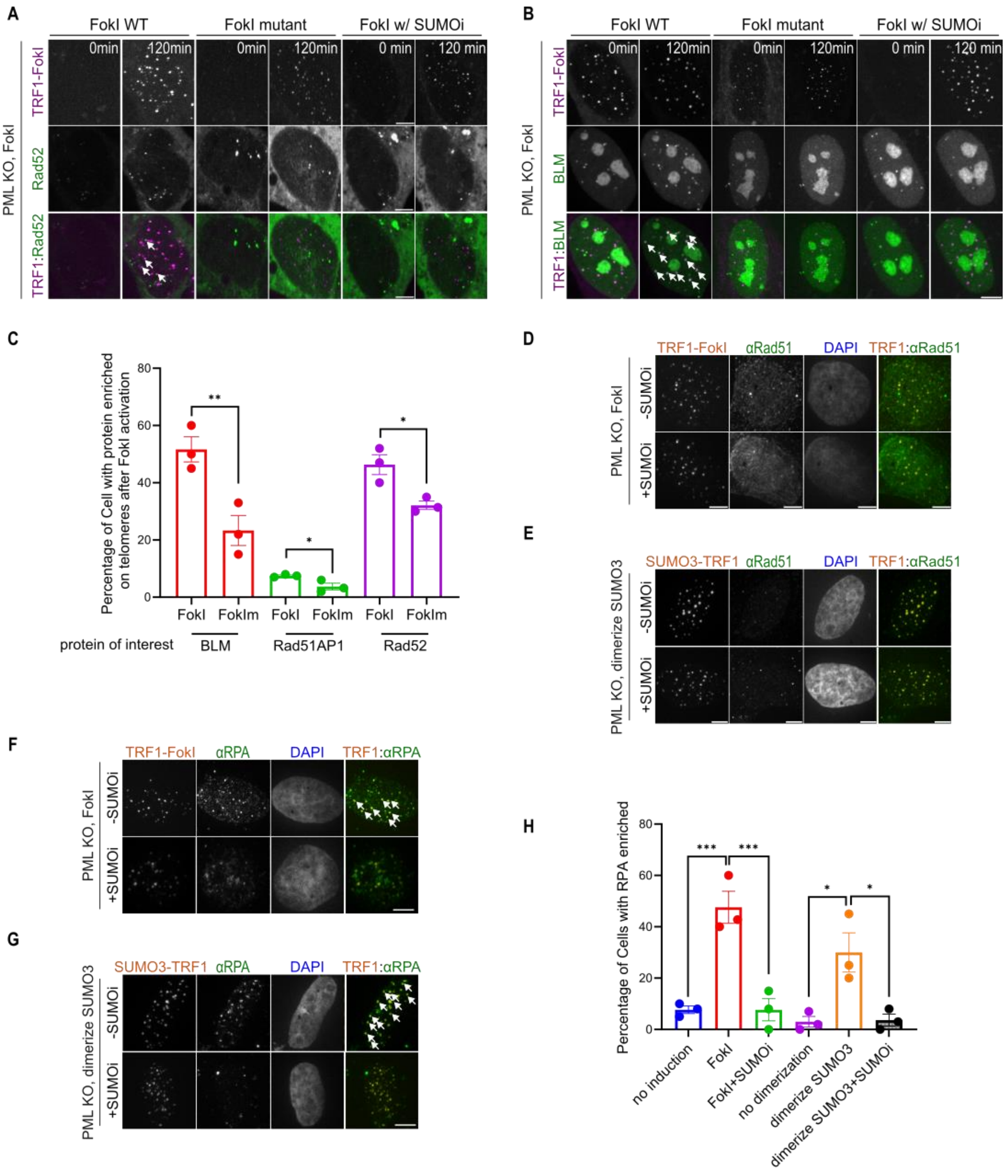


**Fig. S7. Localization of DNA repair factors to telomeres after inducing FokI or recruiting SUMO3 to PML KO telomeres. (A)** Representative images and **(C)** quantification of Rad52 telomeric localization in PML KO cells expressing mCh-TRF1-FokI or enzymatically dead mutant TRF1-FokI-D450A and GFP-Rad52, with 4-OHT added after first time point**. (B)**

Representative images and **(C)** quantification of BLM telomeric localization in PML KO cells expressing mCh-TRF1-FokI or enzymatically dead mutant TRF1-FokI-D450A and GFP-Rad52, with 4-OHT added after first time point. Each dot represents one experiment, three independent experiments, more than 41 cells in each group. **(D, F)** Representative images of PML KO cells with 6-hour FokI activation and stain for Rad51 or RPA, with or without 1 μM SUMOi for 2 days. **(E, G)** Representative images of PML KO cells expressing 3xHalo-TRF1, mCherry-eDHFR-SUMO3 with 6-hour dimerization and stain for Rad51 or RPA, with or without 1 μM SUMOi for 2 days. White arrows indicate RPA localization at telomeres. **(H)** Quantification of RPA localized to telomeres after inducing DNA damage or recruiting SUMO3 to PML KO telomeres for 6 hours. Each dot represents one independent experiment, three independent experiments, more than 32 cells in each group. Scale bars, 5 μm.

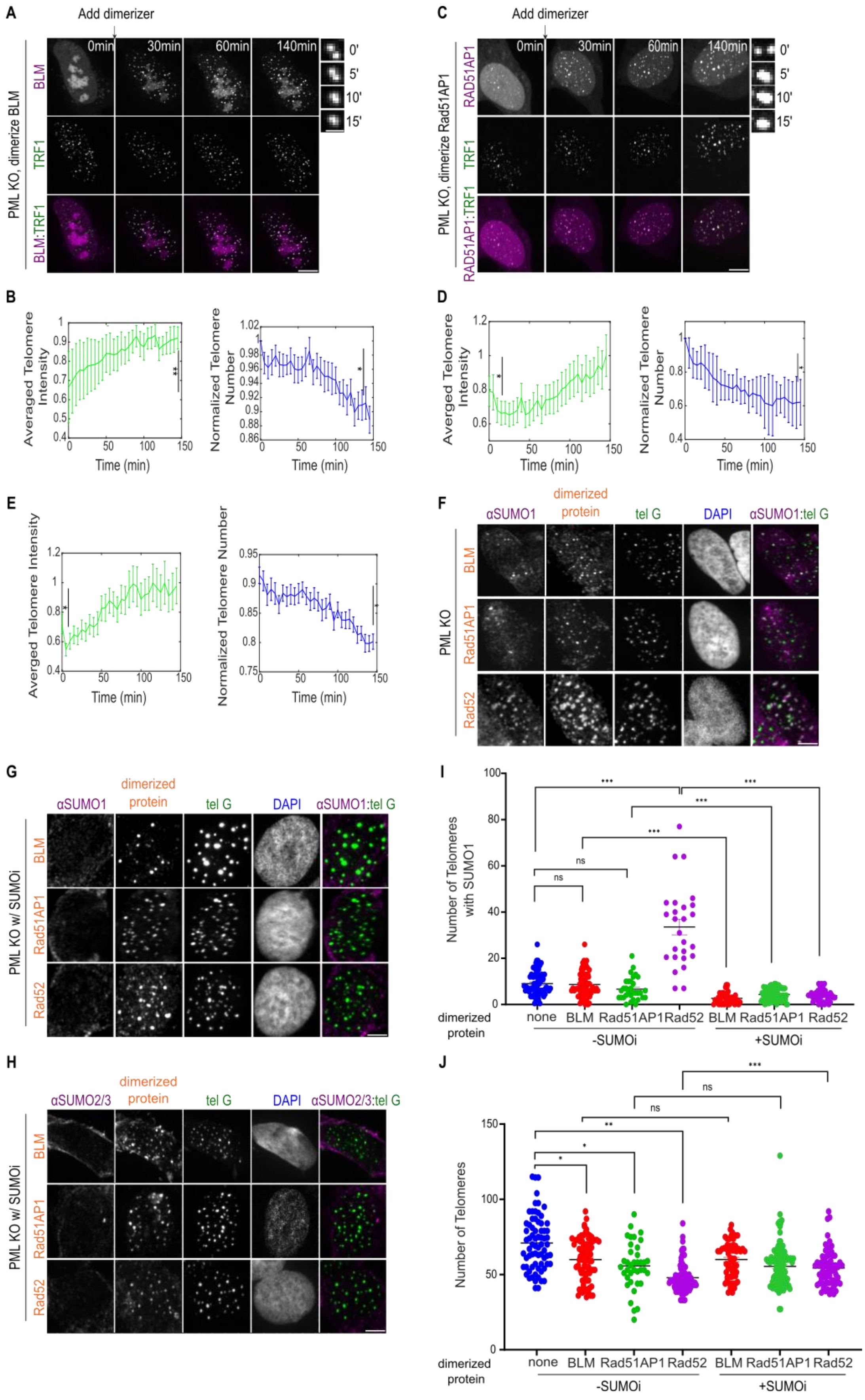
A
Add dimerizer
0min
30min
60min
140min
0'
5'
10'
15'
PML KO, dimerize BLM
BLM
TRF1
BLM:TRF1
C
Add dimerizer
0min
30min
60min
140min
0'
5'
10'
15'
PML KO, dimerize Rad51AP1
RAD51AP1
TRF1
RAD51AP1:TRF1
B
Averaged Telomere Intensity
Time (min)
Normalized Telomere Number
Time (min)
D
Averged Telomere Intensity
Time (min)
Normalized Telomere Number
Time (min)
E
Averged Telomere Intensity
Time (min)
Normalized Telomere Number
Time (min)
F
αSUMO1
dimerized protein
tel G
DAPI
αSUMO1:tel G
PML KO
BLM
Rad51AP1
Rad52
G
αSUMO1
dimerized protein
tel G
DAPI
αSUMO1:tel G
PML KO w/ SUMOi
BLM
Rad51AP1
Rad52
I
Number of Telomeres with SUMO1
dimerized protein
none
BLM
Rad51AP1
Rad52
BLM
Rad51AP1
Rad52
-SUMOi
+SUMOi
H
αSUMO2/3
dimerized protein
tel G
DAPI
αSUMO2/3:tel G
PML KO w/ SUMOi
BLM
Rad51AP1
Rad52
J
Number of Telomeres
dimerized protein
none
BLM
Rad51AP1
Rad52
BLM
Rad51AP1
Rad52
-SUMOi
+SUMOi

**Fig. S8. SUMO enrichment after dimerizing DNA repair factors to telomeres in PML KO cells. (A)** Representative images of PML KO cells after dimerizing mCh-eDHFR-BLM to 3xHalo-GFP-TRF1 at indicated time points. Zoomed-in images show a fusion event of TRF1 foci. **(B)** Telomere sum intensity and telomere number per cell after adding the dimerizer (telomere numbers are normalized by the number at the first time point for each cell, 23 cells per group, three independent experiments, two-tailed unpaired *t-test*). **(C)** Representative images of PML KO cells after dimerizing mCh-eDHFR-Rad51AP1 to 3xHalo-GFP-TRF1 at indicated time points. Zoomed-in images show a fusion event of TRF1 foci. **(D)** Telomere sum intensity and telomere number per cell after adding the dimerizer. (telomere numbers are normalized by the number at the first time point for each cell, 25 cells per group, three independent experiments). **(E)** Telomere sum intensity and telomere number per cell after dimerizing mCh-eDHFR-Rad52 to 3xHalo-GFP-TRF1. (telomere numbers are normalized by the number at the first time point for each cell, 20 cells per group, three independent experiments). **(F)** Representative images of SUMO1 localization at telomeres in PML KO cells after dimerizing mCh-eDHFR-BLM/Rad51AP1/Rad52 to 3xHalo-TRF1. **(G)** Representative images of SUMO1 and **(H)** SUMO2/3 localization at telomeres and **(I)** quantification in PML KO cells after dimerizing mCh-eDHFR-BLM/Rad51AP1/Rad52 to 3xHalo-TRF1 with 1 μM SUMOi for 2 days. **(J)** Number of telomeres in PML KO after dimerizing Rad52/BLM/Rad51AP1 to telomeres for 6 hours, with or without 1 μM SUMO inhibitor for 2 days. Each dot represents one cell, three independent experiments, 76 cells in each group. Scale bars, 5 μm or 1 μm for the zoomed-in images.

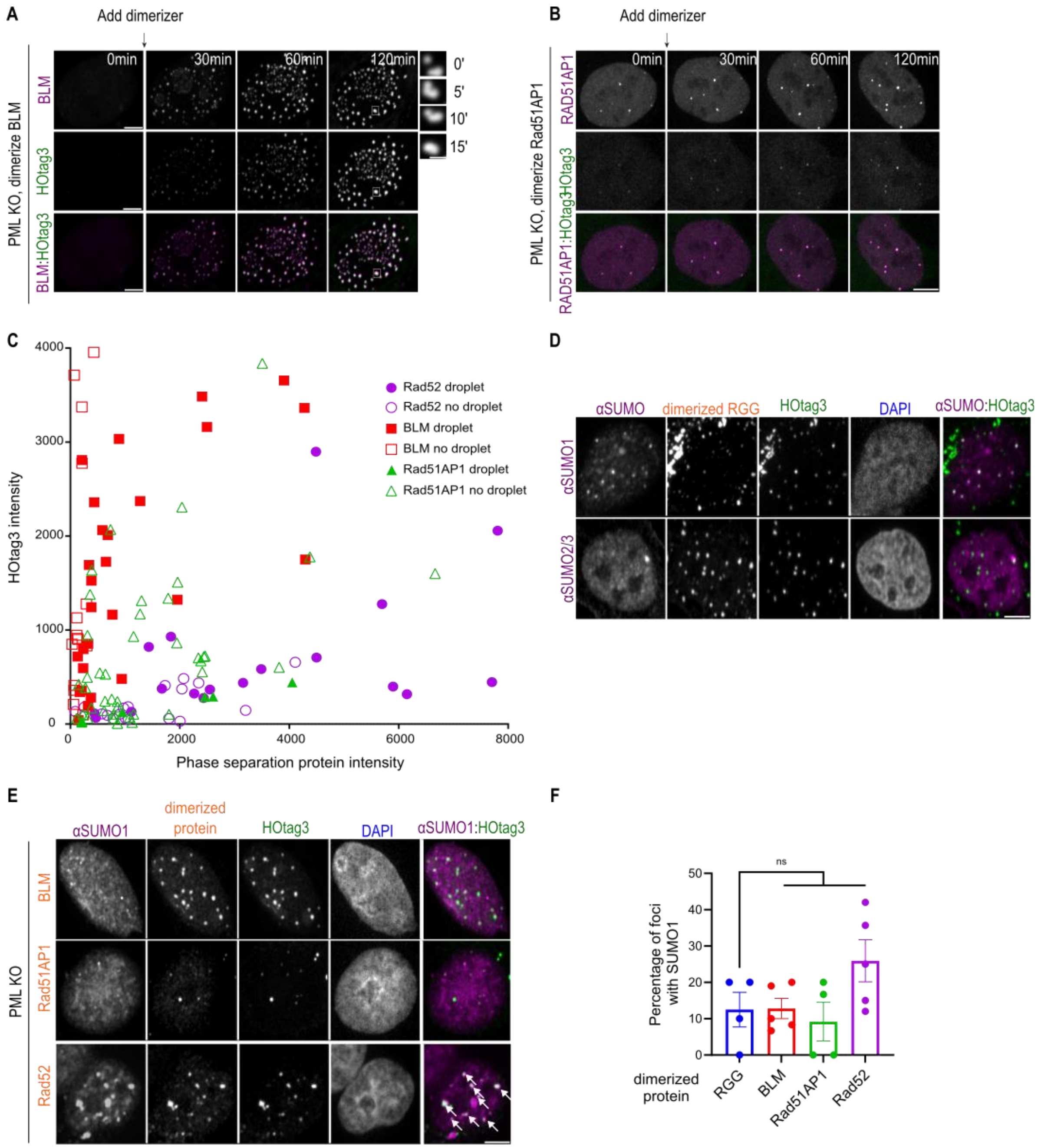


**Fig. S9. Condensate formation and SUMO enrichment after dimerizing DNA repair factors to HOtag3 in PML KO cells. (A)** Representative images of PML KO cells after dimerizing mCh-eDHFR-BLM to 3xHalo-GFP-HOtag3 at indicated time points. Inset Zoomed-in images show a fusion event. **(B)** Representative images of PML KO cells after dimerizing mCh-eDHFR-Rad51AP1 to 3xHalo-GFP-HOtag3 at indicated time points. **(C)** Phase diagram of BLM/Rad51AP1/Rad52 droplet formation. Intensities are the mean intensity in cells before dimerization. Each symbol represents one cell, three independent experiments, 30 cells in each group. **(D)** Representative images of SUMO1/2/3 localization in foci in PML KO cells after

dimerizing RGG-mCh-eDHFR-RGG to 3xHalo-GFP-HOtag3. **(E)** Representative images and **(F)** quantification of SUMO1 localization in foci in PML KO cells after dimerizing mCh-eDHFR-BLM/Rad51AP1/Rad52 to 3xHalo-GFP-HOtag3. White arrows indicate co-localization. Each dot represents one experiment, three independent experiments, more than 33 cells in each group. Scale bars, 5 μm or 1 μm for the zoomed-in images.

221

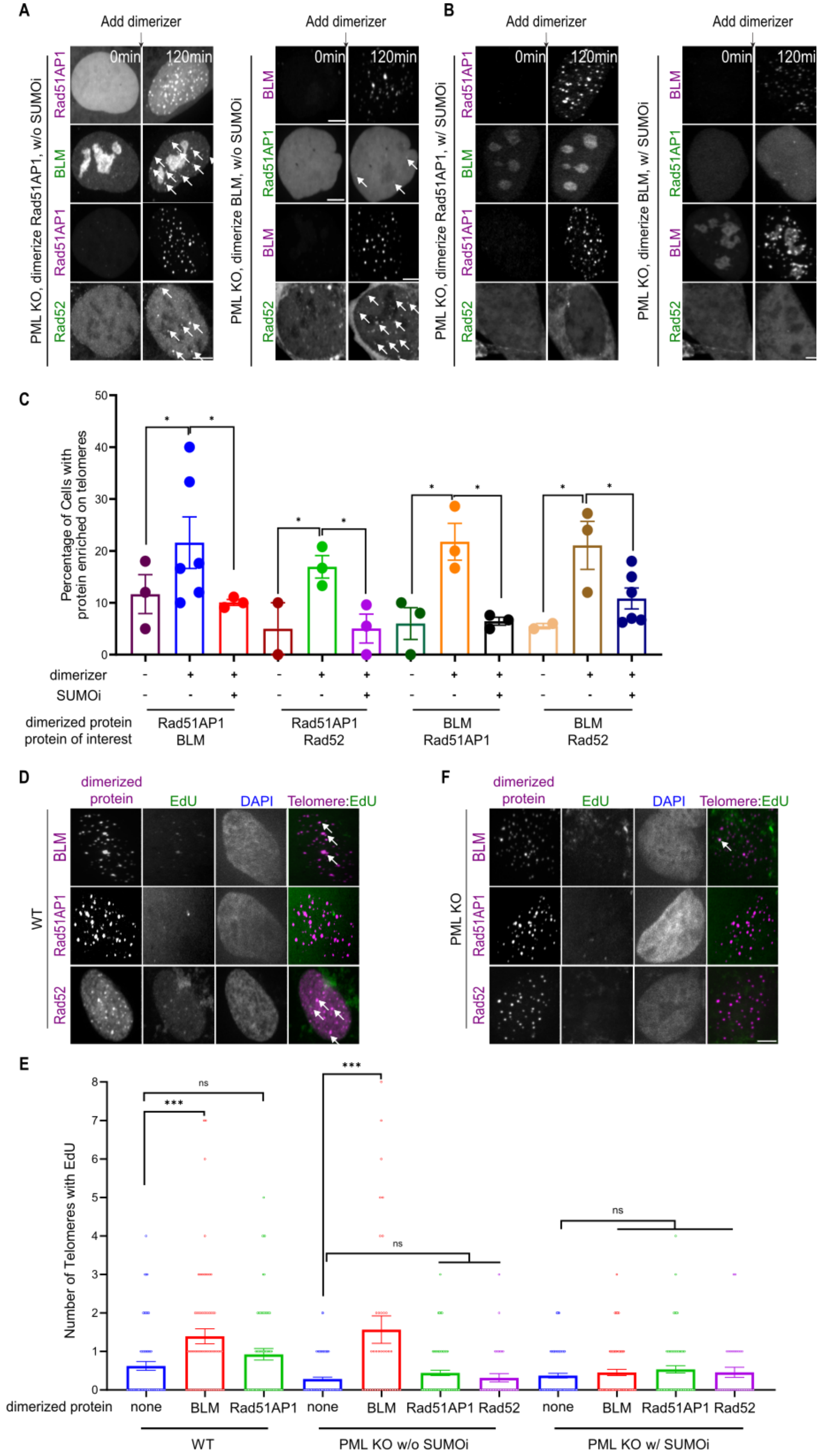
A
Add dimerizer
0min
120min
PML KO, dimerize Rad51AP1, w/o SUMOi
Rad51AP1
BLM
Rad51AP1
Rad52
PML KO, dimerize BLM, w/o SUMOi
BLM
Rad51AP1
BLM
Rad52
B
PML KO, dimerize Rad51AP1, w/ SUMOi
PML KO, dimerize BLM, w/ SUMOi
C
Percentage of Cells with protein enriched on telomeres
dimerizer
SUMOi
dimerized protein
protein of interest
Rad51AP1
BLM
Rad51AP1
Rad52
BLM
Rad51AP1
BLM
Rad52
D
dimerized protein
EdU
DAPI
Telomere:EdU
WT
BLM
Rad51AP1
Rad52
F
PML KO
E
Number of Telomeres with EdU
ns
***
dimerized protein
none
BLM
Rad51AP1
none
BLM
Rad51AP1
Rad52
none
BLM
Rad51AP1
Rad52
WT
PML KO w/o SUMOi
PML KO w/ SUMOi

**Fig. S10. Importance of SUMO for mutual enrichment of repair factors and telomere DNA synthesis. (A, B)** Representative images and **(C)** quantification of protein localization to telomeres in PML KO cells expressing GFP-BLM/Rad51AP1/Rad52 after dimerizing mCh-eDHFR-Rad51AP1/BLM to 3xHalo-TRF1, with or without 1 μM SUMOi for 2 days. White arrows indicate localization at telomeres. Each dot represents one experiment, three independent experiments, more than 35 cells in each group. **(D, F)** Representative images of EdU at telomeres after dimerizing BLM, Rad51AP1, or Rad52 to telomeres in PML KO and WT cells. **(E)** Quantification of EdU at telomeres after dimerizing BLM, Rad51AP1, or Rad52 to telomeres in WT U2OS and PML KO cells with or without 1 μM SUMOi for 2 days. Each dot represents one cell, three independent experiments, more than 36 cells in each group. Scale bars, 5 μm
